# MEDT analysis of mechanism and selectivities in non-catalyzed and lewis acid-catalyzed diels–alder reactions between R-carvone and isoprene

**DOI:** 10.1038/s41598-024-67351-9

**Published:** 2024-07-22

**Authors:** Khadija El Idrissi, Mohamed Abdoul-Hakim, Na’il Saleh, Hocine Garmes, Asad Syed, Mar Ríos-Gutiérrez, Bilal Ahamad Paray, Meenakshi Verma, Abdellah Zeroual, Luis R. Domingo

**Affiliations:** 1 Molecular Modelling and Spectroscopy Research Team, Faculty of Science, Chouaïb Doukkali University, P.O. Box 20, 24000 El Jadida, Morocco; 2 Analytical Chemistry and Environmental Sciences Team, Department of Chemistry, Faculty of Science, University Chouaib Doukkali, El Jadida, Morocco; 3 Department of Chemistry, College of Science, United Arab Emirates University, P.O. Box 15551, Al Ain, United Arab Emirates; 4 Department of Botany and Microbiology, College of Science, King Saud University, P.O. Box 2455, 11451 Riyadh, Saudi Arabia; 5 Department of Organic Chemistry, University of Valencia, Dr. Moliner 50, 46100 Burjassot, Valencia Spain; 6 Department of Zoology, College of Science, King Saud University, PO Box 2455, 11451 Riyadh, Saudi Arabia; 7 University Centre for Research & Development, Department of Chemistry, Chandigarh University, Gharuan, Mohali India

**Keywords:** R-Carvone, MEDT, Lewis acid catalysts, Diels–Alder reactions, ELF, Anti-HIV activity, Density functional theory, Computational models

## Abstract

Within the context of Molecular Electronic Density Theory (MEDT), this study investigates the Diels–Alder reaction among isoprene (**2**) and R-carvone (**1R**) applying DFT simulations, with and without Lewis acid (LA) catalysis. The results show that carvone (**1R**) acts as an electrophile and isoprene (**2**) as a nucleophile in a polar process. LA catalysis increases the electrophilicity of carvone, thereby improving the reactivity and selectivity of the reaction by reducing the activation Gibbs free energy. Parr functions reveal that the C_5_=C_6_ double bond is more reactive than the C_9_=C_10_ double bond, indicating chemoselectivity. The examination of the Electron Localization Function (ELF) reveals high regio- and stereoselectivity, indicating an asynchronous mechanism for the LA-catalyzed DA reaction. Furthermore, it is suggested that cycloadduct **3** has great anti-HIV potential because it exhibits lower binding energies than azidothymidine (AZT) in the docking studies of cycloadducts **3** and **4** amongst a primary HIV-1protein (1A8O plus 5W4Q).

## Introduction

Cycloaddition reactions are versatile and widely used methods for creating carbocyclic compounds, which are essential for many applications in organic chemistry^1^. One of the most important methods for synthesizing carbocyclic compounds of six members is the Diels–Alder (DA) reaction^2–4^. This reaction has found extensive utility in the synthesis of a diverse array of natural products and bioactive compounds. The presence of Lewis acid (LA) catalysts has made the DA reactions involving poor electrophilic ethylenes such as carvone **1** more feasible for total synthesis^5,6^; this cycloaddition reaction constitutes one of the most frequently employed synthetic methods for terpenoid natural products^7^.

Carvone **1** is an oxygenated terpene that occurs naturally in many plants, especially in the mint family. It is one of the main components of essential oils extracted from caraway, dill, spearmint, and lavender^8^. Carvone **1** exists in two stereoisomeric forms, R-(−)-carvone **1R** and S-(+)-carvone **1S**; they form a pair of enantiomers that differ in their biological and sensory effects. Carvone **1** has various applications in the cosmetic, food, and pharmaceutical industries, as it can impart flavor, fragrance, and therapeutic benefits. Moreover, carvone **1** has been investigated for its possible medicinal properties, such as antimicrobial^9^, anticancer^10^, antifungal^11^, and antioxidant activities^12^.

The enantiomers of carvone **1**, both the left-handed and right-handed forms, are widely employed as initial components in the enantioselective synthesis of natural compounds, with a notable focus on the production of sesquiterpenes^13,14^, this application has become prevalent due to the versatility and significance of carvone enantiomers in various chemical and biological processes. Tony K. M. Shing and all.^7^ were able to synthesize the ketonic products between carvone **1R** and isoprene **2** via LA catalyzed DA reactions, with excellent diastereoselectivity and good yields.

**Scheme 1:**
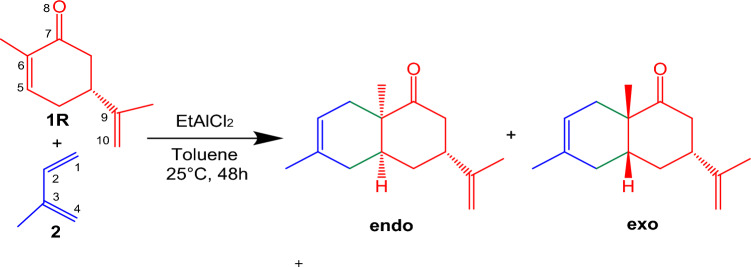
EtAlCl_2_ catalyzed DA reaction of carvone **1R** with isoprene **2**.

EtAlCl_2_ was chosen as a good LA catalyst due to its ease of coordination with the carbonyl oxygen of carvone **1R**, resulting in the easy formation of a bicyclic cycloadduct in a chemo-, regio-, and diastereochemical fashion. (Scheme 1).

The main objective of the present Molecular Electron Density Theory^15^ (MEDT) study is to understand how the EtAlCl_2_ LA catalyst influences the electronic aspects of the DA reaction between carvone **1R** and isoprene **2**, particularly its mechanism and selectivity. To investigate the changes in chemical bonds along the DA reaction, a topological analysis of the electron localization function (ELF)^16^. Concurrently, molecular docking studies were conducted for both the major product P-n-1 and the minor product P-x-2, as observed experimentally, against HIV, using PDB ID: 5W4Q and 1A8O. This analysis aimed to forecast the effect of stereochemistry on the affinity and pharmacological properties of the compounds, comparing them with Azidothymidine.

## Computational details

DFT calculations were performed by using the B3LYP functional^17,18^ together with the 6–311++ G(d, p) basis set, employing the Gaussian 16 software package^19^. Theoretical reactivity indices^20,21^, were obtained by using the equations outlined in reference 21. Within the DFT framework various reactivity indices, encompassing the electronic chemical potential μ, chemical hardness η, global electrophilicity ω^22^, and nucleophilicity *N*^23,24^ were evaluated at the B3LYP/6-31G(d) level. These indices were estimated based on the energies of the frontier molecular orbitals HOMO and LUMO, denoted as H and L, through the following expressions: $\upmu \simeq \frac{({\upvarepsilon }_{H}+{\upvarepsilon }_{L})}{2}$, η ≃ $({\upvarepsilon }_{L}-{\upvarepsilon }_{H})$, $\upomega \simeq \frac{{\upmu ^{2} }}{2\eta }$ . The empirical nucleophilicity *N* index can be expressed using the formula *N* = E_HOMO_(Nu) − E_HOMO_(TCE), where TCE represents tetracyanoethylene^23^. To determine the electrophilic ${P}_{k}^{+}$ and nucleophilic ${P}_{k}^{-}$ Parr functions^25^, the atomic spin densities (ASD) of the radical anion and the radial cation of the reactants were analyzed.

The ELF^16^ was computed using the Multiwfn software^26^. Additionally, a natural bond orbital (NBO) analysis^27^ was conducted to provide further insights into the results.

The global electron density transfer (GEDT)^28^ was calculated by summing the natural atomic charges (q) for the atoms in the transition state structures (TSs) of each framework, as determined by a natural population analysis (NPA)^29,30^.

The intrinsic reaction coordinate (IRC) was used to examine the transition state (TS) associated with the corresponding minimum on the potential energy surfaces^31,32^.

### Ethics approval

The manuscript is prepared in compliance with the Ethics in Publishing Policy as described in the Guide for Authors.

### Consent to participate

The manuscript is approved by all authors for publication.

## Results and discussion

The present MEDT^15^ study is organized into six specific sections: (1) the initial section encompasses an ELF topological analysis of the electronic structure of the reactants; (2) in the second section the theoretical reactivity indices for the reactants are examined; (3) the third section is dedicated to investigating competitive reaction paths associated with the non-catalyzed and LA-catalyzed DA reactions outlined in Scheme 1; (4) the fourth section introduces a Bond Evolution Theory^33^ (BET) study, which focuses on the more favorable reaction path in the DA reaction between carvone **1R** and isoprene **2**, both in the presence and absence of the LA catalyst; (5) in the fifth section, an analysis of ELF compares the preferred DA reaction between carvone **1R** and isoprene **2**, considering both the presence and absence of an EtAlCl_2_ LA catalyst; finally, (6) the sixth section explores molecular docking experiments that investigate the activities of the CAs against the main proteases associated with HIV-1 infection, namely, 5W4Q and 1A8O.

### ELF topological analysis of the electronic structures of carvone 1R, LA:carvone complex 1R:LA, and isoprene 2

**Figure 1:**
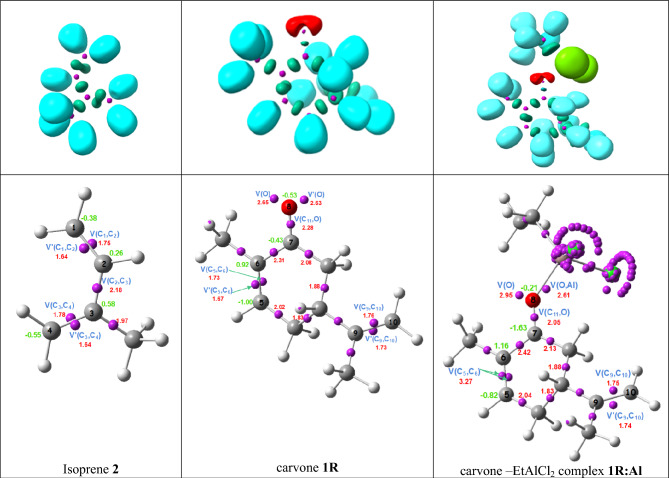
Displays the ELF localization domains of isoprene 2, carvone 1R and LA: carvone complex 1R:LA using the B3LYP/6-311++ G (d,p) method. These domains are revealed with an ELF isosurface value of 0.75, and the populations (red values) and natural atomic charges (green value) for each compound are shown in (e).

Figure 1 provides information on the ELF valence attractor locations, ELF localization domains, and population results for carvone **1R**, LA:carvone complex **1R**:EtAlCl_2_ (**1R:Al**), and isoprene **2**. ELF topological analysis of isoprene **2** in Fig. 1, shows the presence of six disynaptic basins. Notably, the C1–C2 and C3–C4 bonding regions exhibit two pairs of V(Cx,Cy) disynaptic basins with a total population of 3.39 e and 3.42 e, respectively, which correspond to two depopulated C–C double bonds, and a V(C2,C3) disynaptic basin integrating 2.18 e, corresponding to a C–C single bond.

On the other hand, the ELF topological analysis of carvone **1R** shows the presence of two disynaptic basins, V(C5,C6) and Vʹ (C5,C6), integrating a total populations of 3.40 e, a V(C7,O8) disynaptic basin integrating 2.28 e, two monosynaptic basins, V(O1) and Vʹ (O1), integrating a total of 5.28 e, and two disynaptic basins, V(C9,C10) and Vʹ (C9,C10), integrating a total populations of 3.49 e.

Coordination of the EtAlCl_2_ LA catalyst to the O8 oxygen of carvone **1R** slightly modifies the C=C–C=O framework of the LA:carvone complex** 1R:LA**. Thus, the electron density population of the conjugated C5-C6 double bond decreases from 3.40 to 3.27 e, while that of the C9-C10 double bond remains relatively constant with a total electron density population of 3.49 e.

### Analysis of the reactivity indices at the GS of the reagents

Numerous studies devoted to DA reactions have shown that the analysis of the reactivity indices is a powerful tool to predict and understand the reactivity in polar and ionic organic reactions^34^.

The Table 1 displays the electronic chemical potentials μ, chemical hardness η, the electrophilicity ω and nucleophilicity *N* indices for carvone **1R**, isoprene **2**, plus LA:carvone complex **1R:LA**.

**Table 1:** B3LYP/6-31G(d) global properties, specifically, electronic chemical potential, chemical hardness, electrophilicity and nucleophilicity in eV of the reagents **1R**, LA complex **1R:Al**, then **2**.

	μ	η	ω	*N*
LA:carvone complex** 1R:LA**	− 4.88	3.66	3.25	2.42
Carvone **1R**	− 3.84	5.23	1.41	2.67
Isoprene **2**	− 3.30	5.77	0.94	2.94

In comparison to carvone **1R** (μ = − 3.84 eV) and LA:carvone complex **1R:Al** (μ = − 4.88 eV), isoprene **2** exhibits a notably lower electronic chemical potential (μ = − 3.30 eV)^35^. This data strongly indicates that a GEDT from isoprene **2** to carvone **1R** and LA:carvone complex **1R:Al** will occur along the these DA reactions of forward electron density flux (FEDF)^35,36^.

The electrophilicity ω^22^, and nucleophilicity *N*^23,24^ indices of carvone **1R** are 1.41 eV and 2.67 eV, respectively, being classified as a moderate electrophile and a moderate nucleophile within the corresponding scales^23^. Coordination of the EtAlCl_2_ Lewis acid to the O8 oxygen of carvone **1R** notably increases the electrophilicity ω index of the LA:carvone complex **1R:Al**, ω = 3.25 eV, and slightly decreases it nucleophilicity *N* index to 2.42 eV. Thus, LA:carvone complex **1R:Al**, with a ω > 3.00 eV, is classified as a superelectrophile^36^. On the other hand, the electrophilicity ω and nucleophilicity *N* indices of isoprene **2** are 0.90 eV and 2.94 eV, respectively, being classified as a moderate electrophile and in the borderline of strong nucleophile within the corresponding scales.

Consequently, it is expected that while the polar DA reaction between carvone **1R** and isoprene **2** will have some polar character, that involving LA:carvone complex **1R:Al** will have high polar character, showing a high acceleration^37^.

Recent studies on polar cycloaddition reactions have generally shown that the preferred regioselective pathways often involve a two-center interaction between the most electrophilic site of one reactant and the most nucleophilic site of the other^38^. As indicated in previous studies, the analysis of the electrophilic ${P}_{k }^{+}$ and nucleophilic ${P}_{k }^{-}$ Parr functions^25^, arising from the surplus spin electron density transferred via the GEDT^28^ mechanism from the nucleophile to the electrophile, have proven to be the most precise and illuminating tools for evaluating local reactivity in polar and ionic reactions.

**Figure 2:**
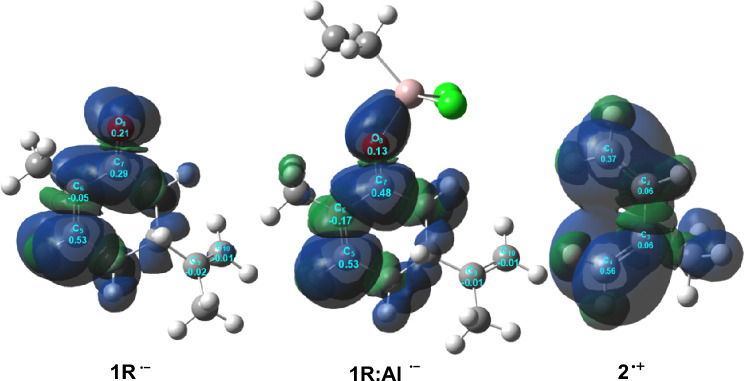
3D representations of the Mulliken atomic spin densities of the radical anion of carvone **1R** and LA:carvone complex **1R:Al**, and those of the radical cation of isoprene **2**, together with the electrophilic ${P}_{k }^{+}$ Parr functions of **1R** and LA:carvone complex **1R:Al** and the nucleophilic ${P}_{k }^{-}$ Parr functions of with isoprene **2**.

Figure 2 illustrates that the carbon atom C4 of isoprene **2** corresponds with the highest nucleophilic center (${P}_{C4}^{-}$ = 0.56) of this species, while the electrophilic ${P}_{k}^{+}$ Parr functions for carvone **1R** and the LA:carvone complex **1R:Al** indicate that C5 exhibits the highest electrophilic center (${P}_{k}^{+}=0.53$). Hence, in agreement with experimental findings, the preferred electrophile–nucleophile interaction in these polar DA reactions occurs along the two-center interactions involving carbon atom C4 of isoprene **2** and carbon atom C5 of carvone **1R** and the LA: carvone complex **1R:Al**. The electrophilic ${P}_{k}^{+}$ Parr functions of the C9 and C10 carbons of **1R** and **1R:Al** (${P}_{k}^{+}<0$) indicate that these two unsaturated carbons are electrophilically deactivated. Consequently, the corresponding polar DA reactions will be completely regio- and chemoselective.

### Exploring the reactions paths associated with in the DA reactions of carvone 1R and LA:carvone complex 1R:Al with isoprene 2

**Scheme 2:**
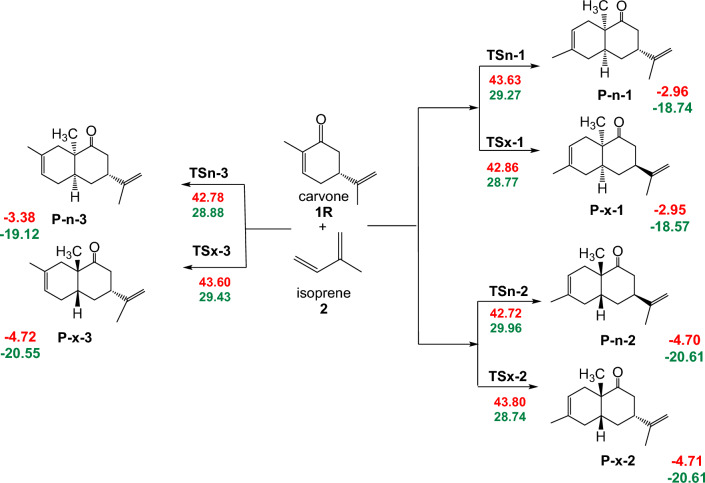
Competitive reaction paths were calculated at a temperature of 25 °C, in the presence of toluene and a pressure of 1 atm for the non-catalyzed DA reactions between R-carvone **1R** and isoprene **2** (the energies are in kcal/mol). (The red values are the free energies, while green values are enthalpies value).

**Scheme 3:**
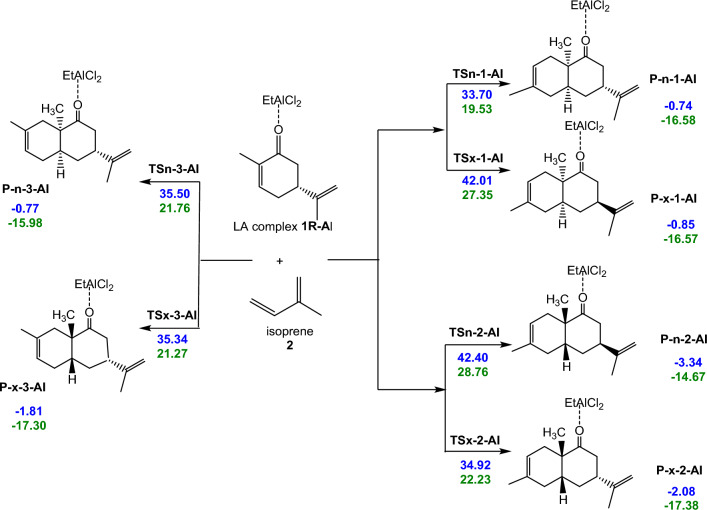
Competitive reaction pathways for were calculated at a temperature of 25 °C, in the presence of toluene and a pressure of 1 atm the LA catalyzed DA reactions of LA: carvone complex **1R:Al** with isoprene **2** (the energies are in kcal/mol) (The value in blue are the free energies, while green value are enthalpies).

Initially, potential energy surface of the reaction paths associated in the DA reactions of carvone **1R** and LA:carvone complex **1R:Al** with isoprene **2** were investigated. The polar DA reactions are *endo* stereoselective, only the endo/exo stereoselectivity for the more favorable regioisomeric reaction paths were studied (see Schemes 2 and 3). These reaction paths involve the stereoselectivity arising from the asymmetry of carvone **1R** and LA:carvone complex **1R:Al** with isoprene **2**, as well as the chemoselective attack of isoprene **2** on the double bonds C5–C6 of carvone **1R** and LA:carvone complex **1R:Al**. Moreover, these C–C double bonds are amenable to stereoselective attacks from either side.

The experimental findings indicate that the LA catalyzed DA reaction of carvone **1R** with isoprene **2** takes place with a complete selectivity, involving solely the C5–C6 double bond, and with a complete regioselectivity, resulting in the production of two stereoisomers associated with the formation of the C1–C6 and C4–C5 single bonds (see Scheme 1). To elucidate the observed regio-, and stereoselectivity, a comprehensive investigation of these six reaction pathways, both in the absence and the presence of the EtAlCl_2_ LA catalyst was conducted.

**Figure 3:**
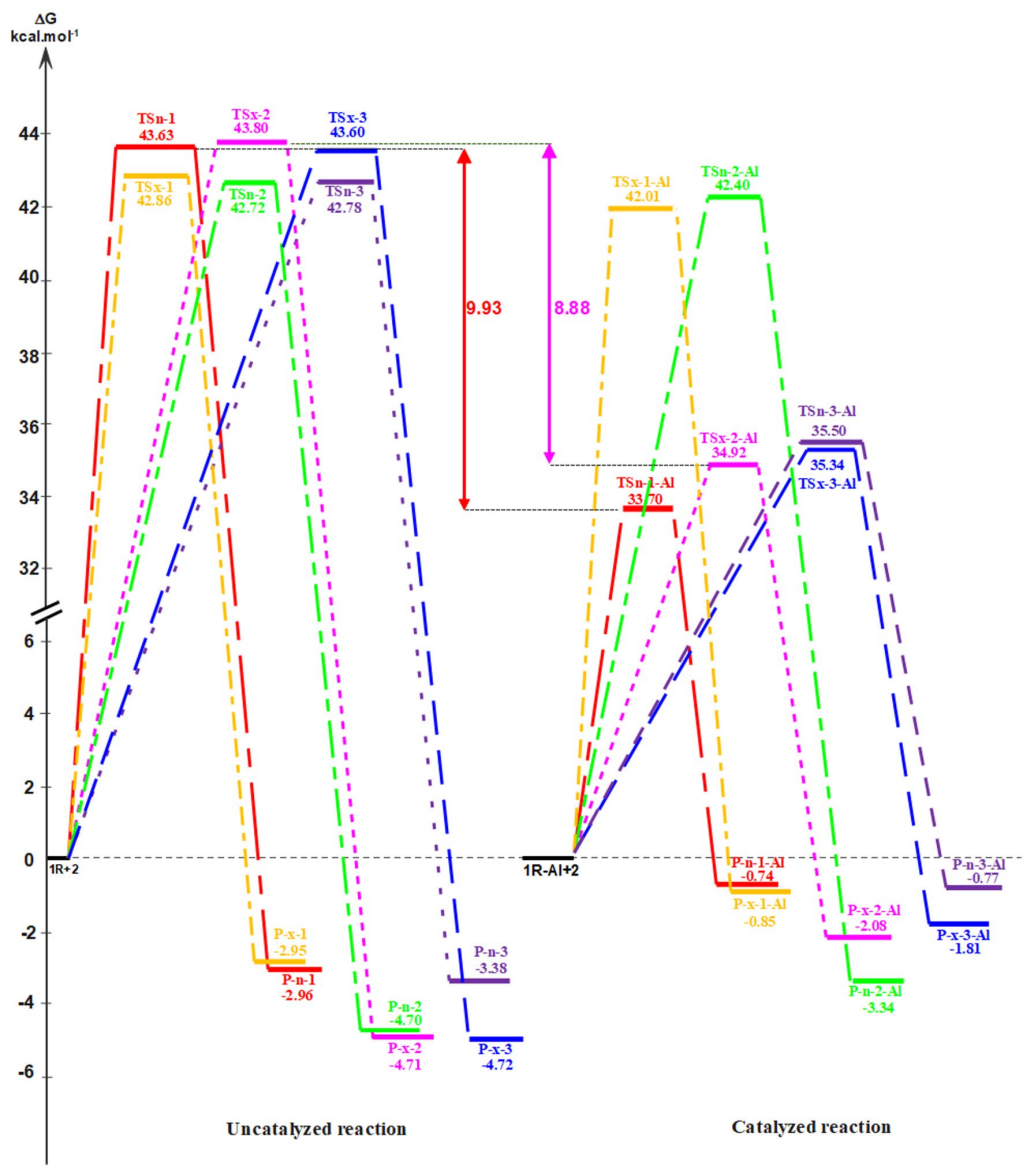
Depicts ΔG of DA reaction between carvone **1R** and isoprene **2** in the presence of toluene at 25 °C, under both non-catalyzed and EtAlCl_2_-catalyzed conditions.

Along each of the six reaction paths only one TS and one CA was characterized for both the non-catalyzed and LA catalyzed DA reactions between carvone **1R** and isoprene **2** (see Schemes 2 and 3), indicating that these DA reactions take place via a one-step mechanism. Figure 3 illustrates the relative Gibbs free energy values for the stationary points associated to the non-catalyzed and LA catalyzed DA reactions of carvone **1R** and isoprene **2** computed in toluene at 25 °C. For detailed thermodynamic data, frequency details, and Cartesian coordinates for the TSs are provided in Supplementary.

Analysis of Tables S1 and Tables S2 highlights the activation enthalpy of the 2 + 4 cycloaddition reaction between R-Carvone (1R) and Isoprene (2), with and without catalyst. The tables provide detailed comparisons of the activation enthalpies (∆H) for different systems. For the TSn-1-Al system, the activation enthalpy without catalyst is 29.27 kcal/mol, while with catalyst it is 19.53 kcal/mol, indicating a significant reduction of 9.74 kcal/mol, suggesting significant catalytic efficiency. In contrast, for the TSx-1-Al system, the reduction is less marked, with the enthalpy of activation falling from 28.77 kcal/mol without catalyst to 27.35 kcal/mol with catalyst, a reduction of 1.42 kcal/mol. For the TSx-2-Al system, the reduction of 6.51 kcal/mol is notable, with activation enthalpy falling from 28.74 kcal/mol without catalyst to 22.23 kcal/mol with catalyst. The TSn-2-Al system shows little catalytic influence, with a reduction of 1.2 kcal/mol. The TSn-3-Al system shows a notable reduction of 7.12 kcal/mol, and finally, the TSx-3-Al system shows a significant reduction of 8.16 kcal/mol. In conclusion, the analysis reveals that the presence of a catalyst reduces the enthalpy of activation in all the systems studied, although the effect varies according to the specific system, with the TSn-1-Al, TSx-2-Al, TSn-3-Al, and TSx-3-Al systems showing notable reductions in enthalpy of activation.

The relationship ΔG = ΔH − TΔS shows that the free enthalpy of activation is influenced by entropy. Negative entropy values (ΔS) indicate that their contribution (TΔS) increases ΔG. Despite this increase, the reduction in ΔH, and therefore entropy ΔS, destabilizes the reaction. Figure 3 shows that the free energies of activation of the two stereoisomeric reaction pathways in the uncatalyzed reaction range from 42.72 to 43.63 kcal/mol for endo TS and from 42.86 to 43.80 kcal/mol for exo TS. The six P-DA reactions are exothermic from 2.95 to 4.72 kcal/mol. The coordination of the EtAlCl_2_ LA catalyst to carvone 1R results in a significant decrease in the activation Gibbs free energies associated with both reactive channels, the values of these energies ranging from 33.70 to 42.40 kcal/mol for the endo TSs, and from 34.92 to 42.01 kcal/mol for the exo ones.

Figure 3 shows a summary of the free energies data. These relative Gibbs free energies show that TSn-1-Al is kinetically more favored than TSn-1 by an average of 9.93 kcal/mol, however the thermodynamic stability of P-n-1-Al is estimated at 1.34 kcal/mol when compared to P-x-2-Al. These findings suggest that the regioisomer P-n-1-Al is obtained due to uncontrolled kinetic factors, while Experimental finding indicate that, P-n-1-Al is the predominant product, with a yield of 92.06%, while P-x-2-Al is obtained with only a yield of 7.94%.

Furthermore, TSx-1-Al (34.92 kcal/mol) is 8.88 lower compared to that of TSx-1 (43.80 kcal/mol), this observation indicates a remarkable enhancement in the efficiency of the reaction in the presence of the catalyst.

This trend is consistent across all transition states, highlighting the catalytic prowess of Et2AlCl3 in facilitating the Diels–Alder reaction between carvone 1R and isoprene 2.

The most favorable reaction path result in the formation of stereoisomer P-n-1-Al as the major product and stereoisomer P-x-2-Al as the minor product, occurring through TSn-1-Al and TSx-2-Al, respectively. These TSs are situated 33.70 kcal/mol (TSn-1-Al) and 34.92 kcal/mol (TSx-2-Al) kcal.mol-1 above the reactants. The activation free energy difference between TSn-1-Al and TSx-2-Al, represented by ΔΔG = 1.22 kcal/mol, signifies significant regioselectivity observed experimentally in this LA-catalyzed 42DC reaction.

**Figure 4:**
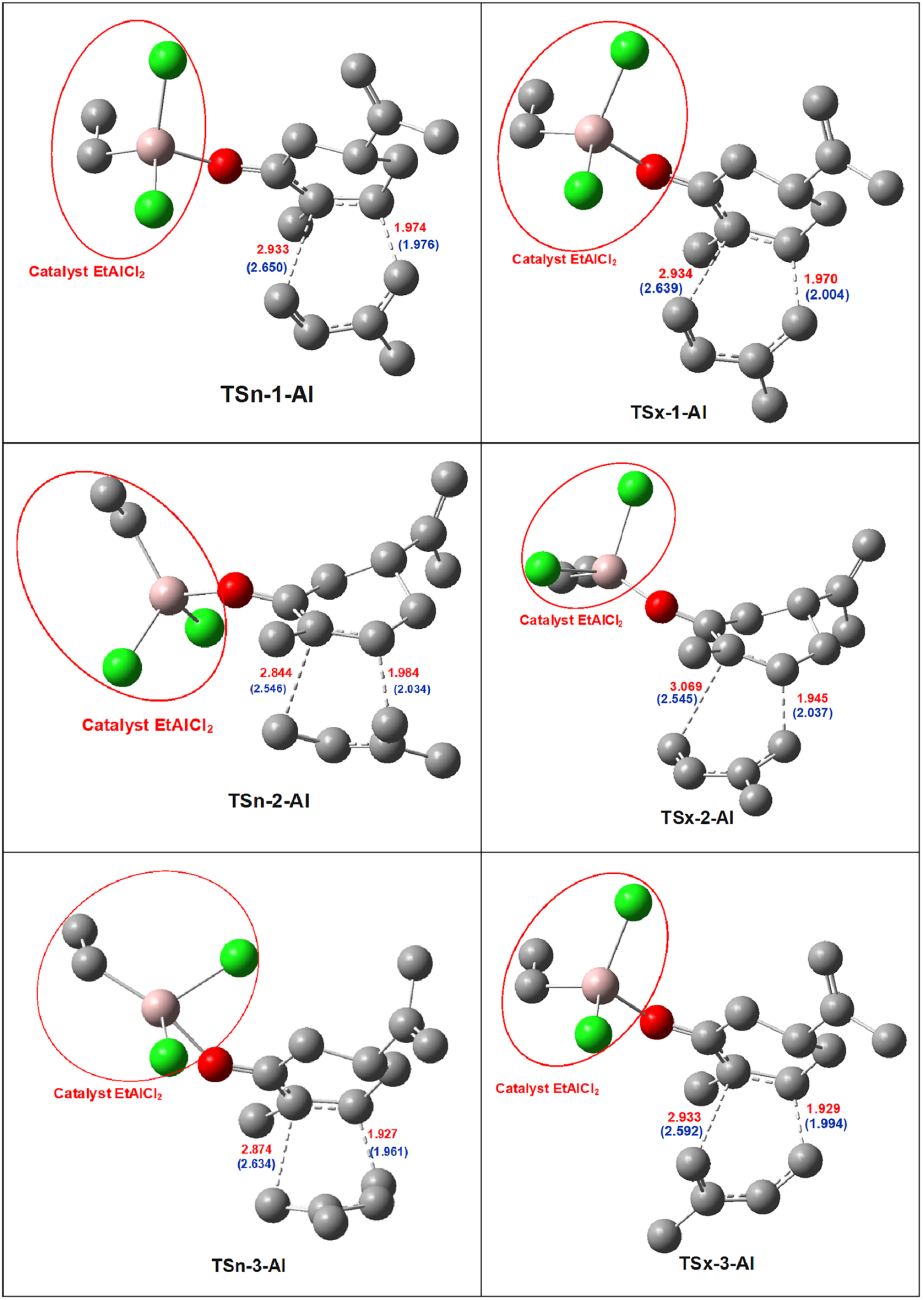
The optimized geometries in toluene of the TSs for the LA catalyzed DA reaction between carvone **1R:Al** and isoprene **2**. Non-catalyzed values are presented in blue, and distances are given in angstroms (Å).

The optimized geometries in toluene of the TSs involved in the LA catalyzed DA reaction of LA: carvone complex **1R:Al** with isoprene **2** are displayed in Fig. 4.

At the TSs involving the C5–C6 double bond of LA:carvone complex **1R:Al**, the distances between the two pairs of interacting carbons are:2.933 Å (C1–C6) and 1.974 Å (C4–C5) at**TSn-1-Al**, 2.934 Å (C1–C6) and 1.970 Å (C4–C5) at **TSx-1-Al**, 2.844 Å (C1–C6) and 1.984 Å (C4–C5) at **TSn-2-Al**, and 3.069 Å (C1–C6) and 1.945 Å (C4–C5) at **TSx-2-Al**, 2.874 Å (C4–C6) and 1.927 Å (C1–C5) at**TSn-3-Al**, 2.933 Å (C4–C6) and 1.929 Å (C1–C5) at **TSx-3-Al**. In the preferred reaction path involving the C5–C6 double bond of LA:carvone complex **1R:Al**, the level of asynchronicity at the TSs varies as follows: 0.95 at **TSn-1-Al**, 0.96 at **TSx-1-Al**, 0.86 at **TSn-2-Al**, 1.12 at **TSx-2-Al**, 0.95 at **TSn-3-Al**, 1.00 at **TSx-3-Al**. In contrast, the asynchronicity at TSs in the absence of LA catalyst is: 0.67 (**TSn-1**); 0.64 (**TSx-1**); 0.51 (**TSn-2**); 0.51 (**TSx-2**), 0.67 (**TSn-3**), 0.60 (**TSx-3**). These geometrical features lead to the conclusion that in this DA reaction, the TSs associated with the catalyzed reaction display more asynchronicity than those associated with the non-catalyzed one These geometrical features lead to the conclusion that in this DA reactions, the TSs associated to the catalyzed reaction are more asynchronicity than those associated with the non-catalyzed one.

The GEDT values computed at the TSs for both non-catalyzed and LA catalyzed DA reactions are presented in Table 2. GEDT values lower than 0.05 e correspond to non-polar processes, while values higher than 0.20 e correspond to highly polar processes. A clear correlation between the calculated GEDT values and the computed activation barriers can be established as described in Tables S1 and Tables S2 in the supplementary information.

**Table 2:** GEDT values for non-catalyzed and LA catalyzed DA reactions, measured in electron units.

Non-catalyzed DA reaction		LA catalyzed DA reaction	
TSn-1	0.15	**TSn-1-Al**	0.34
TSx-1	0.14	**TSx-1-Al**	0.31
TSn-2	0.13	**TSn-2-Al**	0.33
TSx-2	0.13	**TSx-2-Al**	0.35
TSn-3	0.13	**TSn-3-Al**	0.31
TSx-3	0.11	**TSx-3-Al**	0.30

Significant values are in bold.

The GEDT values calculated at the more favorable TSs associated to the attach of isoprene **2** on the attached to the C5–C6 double bond of carvone **1R** have a polar character, with GEDT values less than 0.20 e, while the TSs associated to the attach of isoprene **2** on the C5–C6 double bond of LA:carvone complex **1R-Al**, which present a GEDT > 0.30 e, have a very high polar character as a consequence of the superelectrophilic character of the LA:carvone complex **1R-Al** (see Table 1). The GEDT, which fluxes from isoprene **2** to LA:carvone complex **1R-Al** permits to classify the experimental LA catalyzed DA reaction as the FEDT^35,36^.

### BET study of the molecular mechanisms in the non-catalyzed and LA catalyzed DA reaction involving carvone 1R and isoprene 2

**Figure 5:**
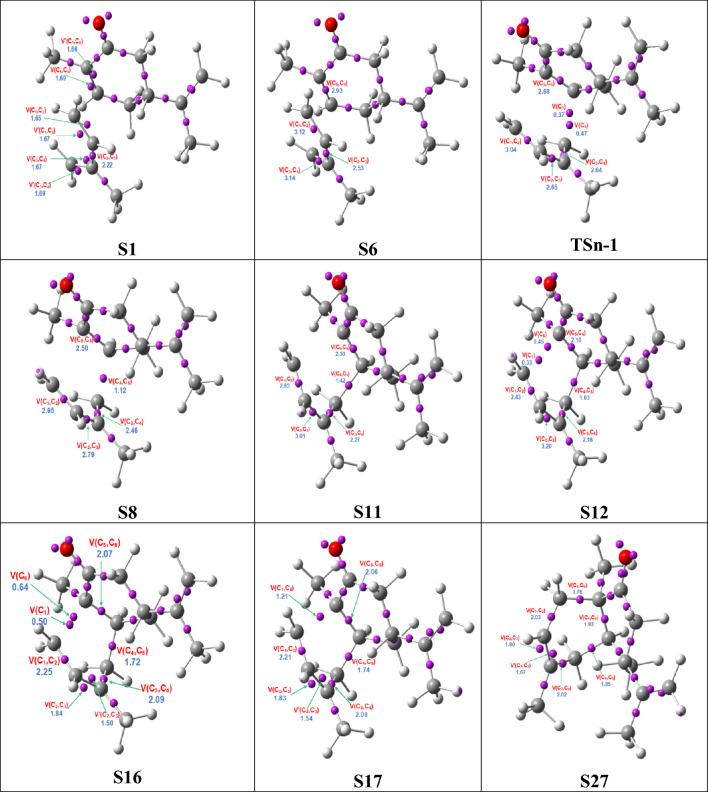
ELF attractors at critical points along the IRC, illustrating the formation of C4–C5 and C1–C6 single bonds in the non-catalyzed DA reaction between carvone **1R** and isoprene **2**.

**Figure 6:**
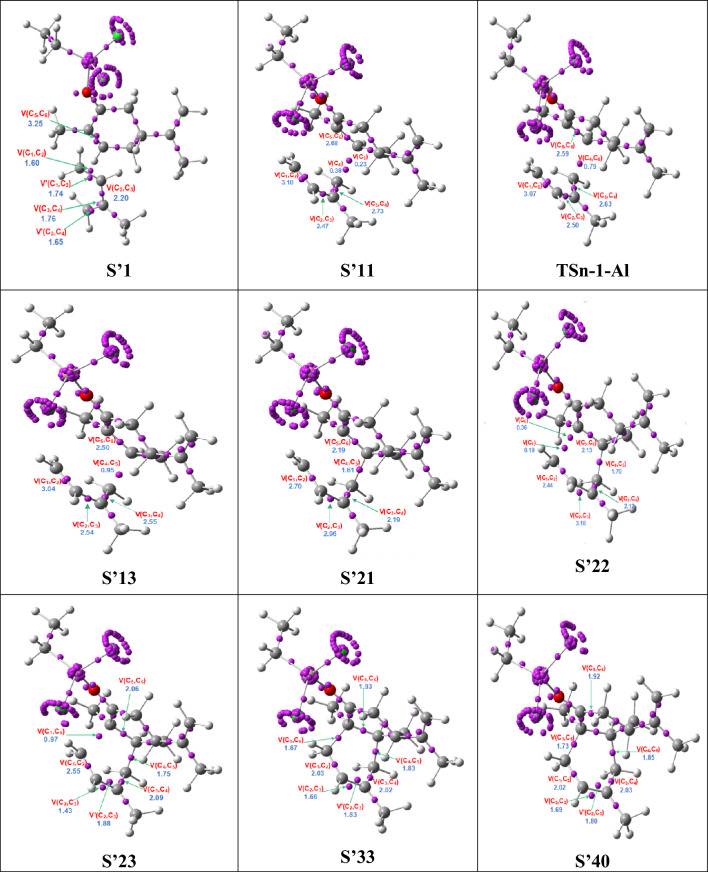
ELF attractors at critical points along the IRC, illustrating the formation of C4–C5 and C1–C6 single bonds in the LA catalyzed DA reaction between carvone **1R** and isoprene **2**.

**Figure 7:**
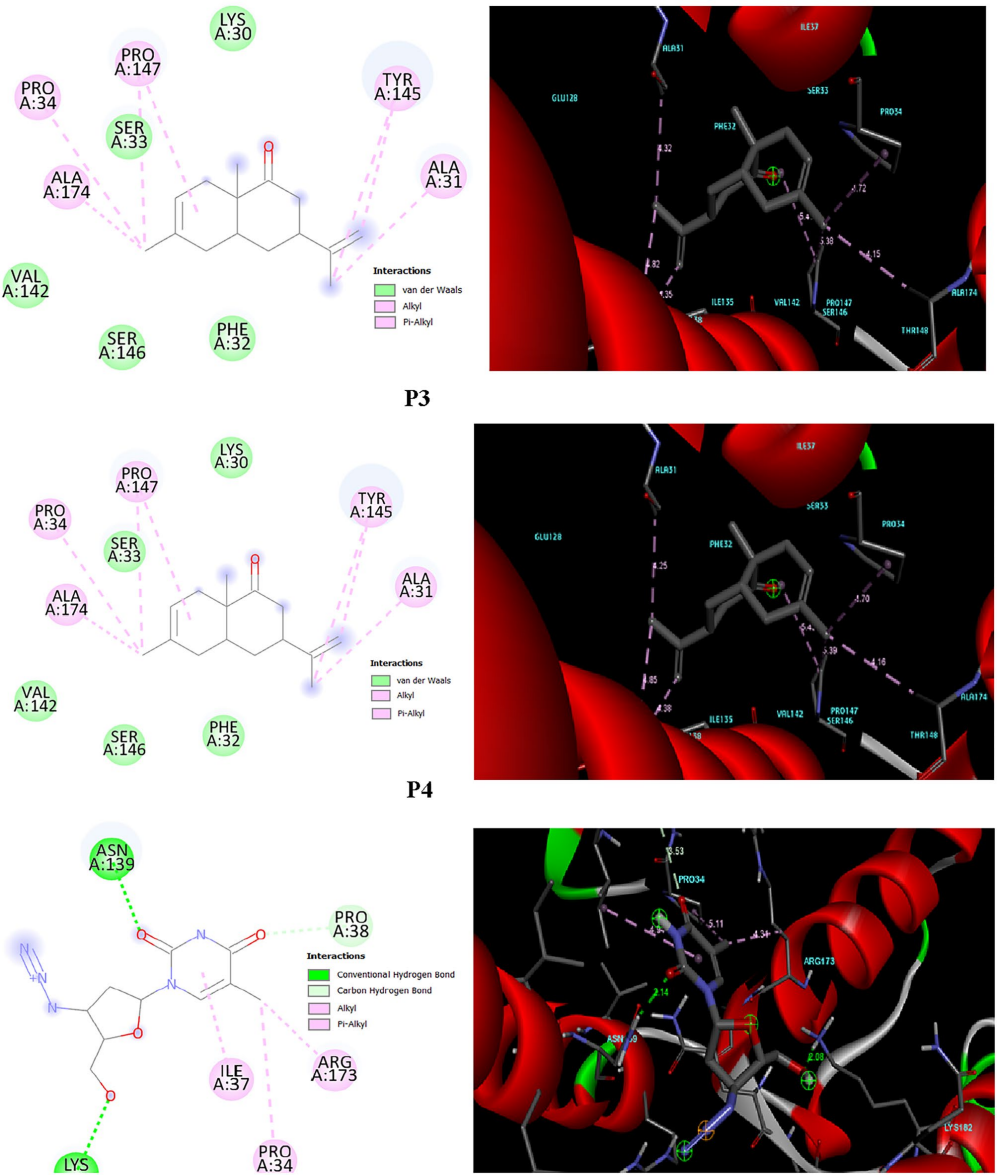
Binding orientation and molecular interactions of the CA3, CA4 and Azidothymidine compounds into the active sites of 5W4Q.

To understand the bonding changes occurring along the most favorable reaction path of the DA reaction involving carvone **1R** and LA:carvone complex **1R:Al** with isoprene **2**, via **TSn-1** and **TSn-1-Al**, respectively, a BET investigation was carried out^33^. This analysis focused on the redistribution of electron density along the selected reaction paths, as illustrated in Figs. 6 and 7. BET is an invaluable tool for elucidating alterations in bonding throughout a reaction path. It offers valuable insights into the nature of electronic changes associated with a particular chemical mechanism^39^. Detailed information about the two BET can be found in Tables S3 and Tables S4 in the Supporting Information. Figures 5 and 6 illustrate the positions of ELF basin attractors for the most relevant structures **SX** involved in the formation of the new C1–C6 and C4–C5 single bonds, in both non-catalyzed and LA catalyzed processes.

The two BET studies provide the following key findings: (1) the formation of the two C–C single bonds occurs in two distinct phases along the IRC, in the non-catalyzed and LA catalyzed DA reactions (2) in the absence of the LA catalyst, a new V(C4) monosynaptic basin, with an initial electron population of 0.19 e, is created at structure **S6**. The population of this monosynaptic basin increases at **TSn-1**. In contrast to the non-catalyzed DA reaction where the C4–C5 single bond is formed after to pass **TSn-1**, in the LA catalyzed reaction, the C–C single bond is formed before to reach **TSn-1-Al**; (3) in both non-catalyzed and LA catalyzed processes, the fusion of two *pseudoradical centers*^40^, characterized by the presence of the V(C4) and V(C5) monosynaptic basins, permit the formation of the first C4–C5 single bond at a distance of 1.88 Å in the non-catalyzed process, after to pass **TSn-1**, and a distance of 1.97 Å in the LA catalyzed one, at the **TSn-1-Al**^28^; (4) in the early stages of the reaction, the C5–C6 double bond in carvone **1R** experiences a gradual reduction in electron population, resulting in a decrease from 3.32e to 1.93e in the absence of the LA catalyst, and from 3.25e to 1.92e when the catalyst is present. These bonding changes are demanded for the subsequent formation of the C5 *pseudoradical* centers. A similar behavior is observed in the C1–C2 and C3–C4 double bonds of isoprene **2**. In particular, in the non-catalyzed DA reaction, the electron population of the C1–C2 double bond decreases from 3.32e to 1.93e, while in the LA catalyzed reaction, it decreases from 3.25e to 1.92e. The electron population also decreases for the C3–C4 double bond, dropping from 3.32e to 1.93e in the non-catalyzed process and from 3.25e to 1.92e in the LA catalyzed reaction; (6) in the LA catalyzed reaction, at structure **Sʹ22**, two monosynaptic basins, V(C1) and V(C6), are simultaneously created, with an electron populations of 0.19 and 0.36 e, respectively (as illustrated in Fig. 6). These V(C1) and V(C6) monosynaptic basins correspond to *pseudoradical* centers located at C1 and C6 carbons, which are demanded for the subsequent creation of the second C1–C6 single bond. Subsequently, at structure **Sʹ23**, both V(C1) and V(C6) monosynaptic basins disappear, making way for the formation of a new disynaptic basin, V(C1,C6), with an initial population of 0.97e (as illustrated in Fig. 6). These changes in electron density indicate that the second C1–C6 single bond has been formed at a C–C distance of 2.08 Å by the coupling of the two C1 and C6 *pseudoradical* centers. In contrast, in the non-catalyzed process, at structure **S17**, a new disynaptic basin V(C1,C6) appears, with an initial electron population of 1.21e (as depicted in Fig. 5). These electronic changes signify the formation of the second single bond C1–C6 at a C–C distance of 2.07 Å through the fusion of the electron densities of the two V(C1) and V(C6) monosynaptic basins, which integrated 0.50 and 0.64e, respectively, at structure **S12**; (6) moving from structures **Sʹ24** to **Sʹ40** in the catalyst reaction, there is a relaxation process leading to the formation of the final CA **3**. This progression aligns with the expected structure for the CA **3**, as depicted in **Sʹ40** in Fig. 6; (7) based on these behaviors, it can be concluded that the LA catalyzed DA reaction follows a high asynchronous, non-concerted molecular mechanism. This mechanism can be defined as a *two-stage one-step* process^41^, as the creation of the second C1–C6 single bond begins after that the formation of the first C4–C5 single bond has been established in 80%.

### Molecular docking

The global population faces the problem of viral infections and the emergence of life-threatening diseases caused by various viruses, including HIV-1^42^. HIV-1, the virus accountable for AIDS (Acquired Immunodeficiency Syndrome), substantially undermines the immune system, leading to numerous health-related complications.

To address this critical issue, molecular docking studies have been conducted, offering the potential to save time, expedite early preclinical research, and significantly reduce costs.

This technique has the capability to become a powerful tool in the development of strategies to combat these diseases and build a robust arsenal against them.

This section details a docking study conducted to explore potential interactions between the examined compounds and the HIV-1 protein. The central aim of this investigation is to gain a more profound insight into how these compounds can bind to and interact with viral protein. Consequently, this allows for a comparative analysis with the Azidothymidine (AZT) target, offering valuable insights into the potential contributions of these compounds in the fields of HIV-1 research and therapy. Ultimately, this study enhances our comprehension of their effectiveness and the mechanisms they employ to combat the viral virus.

Molecular docking studies require that both the ligands (product 3, product 4, and Azidothymidine) and the HIV-1 target (PDB ID: 5W4Q and 1A8O) possess a three-dimensional structure. The three-dimensional structures for 5W4Q and 1A8O were downloaded from the RCSB Protein Data Bank^43,44^. However, for the AZT ligand, its three-dimensional structure was obtained from PubChem, a database that provides comprehensive information, encompassing both chemical data and chemical structures^45^.

Molecular docking simulations were conducted using Auto Dock Tools 1.5.6^46^. In order to determine the molecular binding affinity of the protein–ligand complex, to visualize intricate interactions and elucidate the 2D and 3D structures of the ligand compounds docked with the HIV-1 protease protein (PDB ID: 5W4Q and 1A8O), BIOVIA Discovery Studio Visualizer and PyMol the bioinformatics software were employed^47,48^. Before the compounds were docked with the HIV-1 target, the proteins were preprocessed by eliminating water molecules and incorporating polar hydrogens and Kollman charges using ADT.

In docking investigation utilizing AutoDock Tools, grid boxes with dimensions of (40 Å × 40 Å × 40 Å) were created for both proteins proteases 5W4Q and 1A8O.

Based on the results of the docking studies, the data for two ligands with the HIV-1 protease, as well as a comparison to Azidothymidine as an antiviral drug, are indeed provided in Table 2.

**Figure 8:**
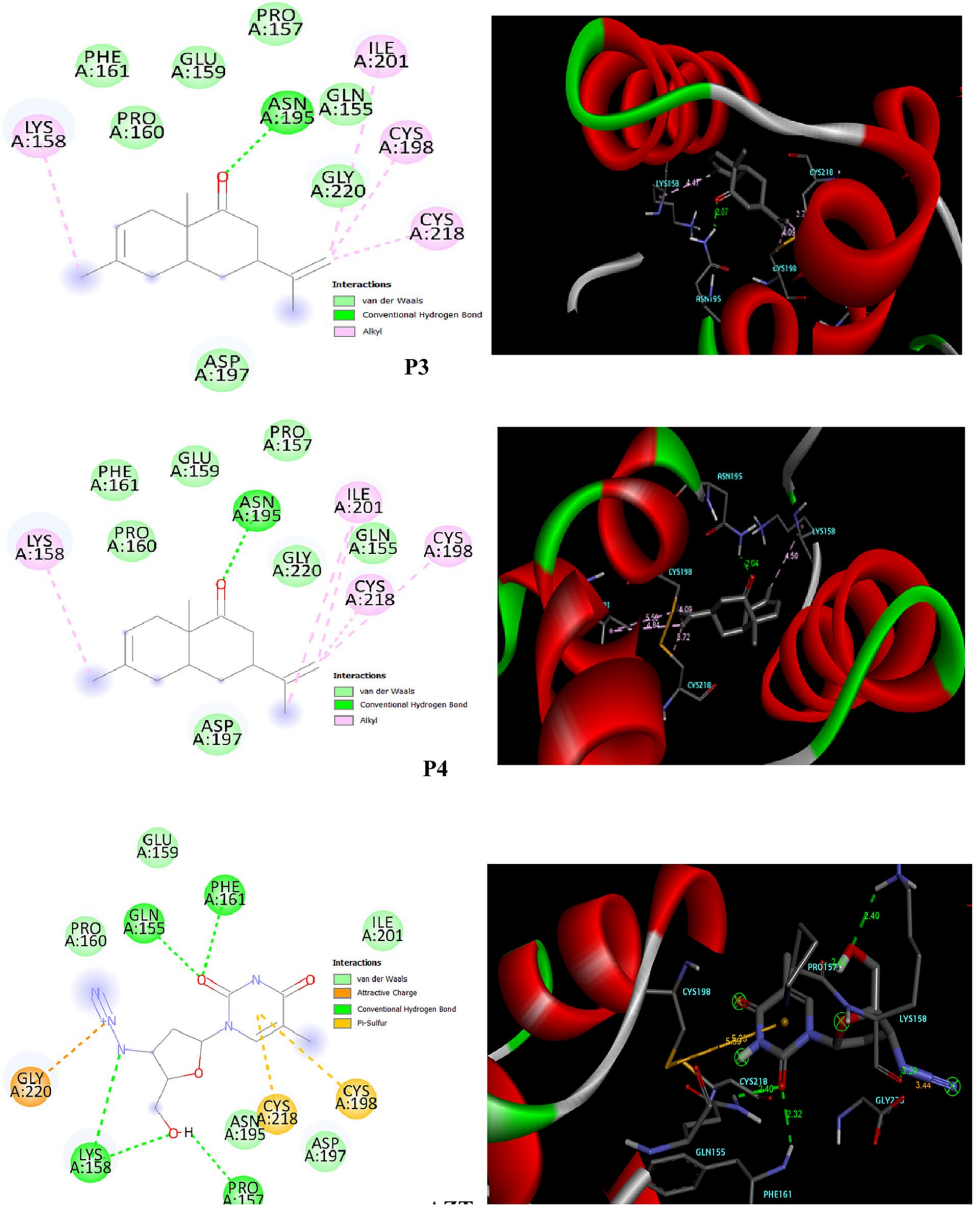
Binding orientation and molecular interactions of the the CA3, CA4 and Azidothymidine compounds into the active sites of 1A8O.

The interactions between the ligands and the macromolecular target (Contrast with AZT) were predicted in both 2D and 3D using BIOVIA Discovery Studio Visualizer, these predictions are presented in Figs. 7 and 8.

According to the data provided in Table 3, the binding energy for the two ligand compounds and the drug AZT with the HIV-1 protein receptor falls within the range of − 5.4 kcal/mol to − 5.7 kcal/mol for 5W4Q and − 5.5 kcal/mol to − 5.7 kcal/mol for 1A8O.

**Table 3:** Molecular docking results of the CA1, CA2 and Azidothymidine comparison.

Name	Product 3 (3R,4aS,8aR)-6,8a-dimethyl-3-(prop-1-en-2-yl)-3,4,4a,5,8,8a-hexahydronaphthalen-1(2H)-one		Product 4 (3R,4aR,8aS)-6,8a-dimethyl-3-vinyl-3,4,4a,5,8,8a-hexahydronaphthalen-1(2H)-one		AZT Azidothymidine	
2D Compound Structure						
3D Compound Structure						
Binding energy						
5W4Q	− 5.7		− 5.6		− 5.4	
1A8O	− 5.5		− 5.5		− 5.7	
Interaction residues-bond distance (Å)						
5W4Q	**ALA174**	4.15	**ALA174**	4.16	**PRO34**	5.11
	**PRO34**	4.72	**PRO34**	4.70	**ILE37**	4.94
	**PRO147**	5.38 5.41	**PRO147**	5.39 5.43	**ARG173**	4.31
	**TYR145**	4.35 4.82	**TYR145**	4.38 4.85	**PRO38**	3.53
	**ALA31**	4.32	**ALA31**	4.25	**ASN139**	2.14
					**LYS182**	2.08
1A8O	**LYZ158**	4.47	**LYZ158**	4.50	**PHE161**	2.32
	**ASN195**	2.07	**ASN195**	2.04	**GLN155**	2.40
	**CYS198**	4.09	**CYS198**	4.09	**PRO157**	2.42
	**CYS218**	3.73	**CYS218**	3.72	**LYS158**	2.40 3.29
	**ILE201**	4.84	**ILE201**	5.50 4.84	**GLY220**	3.44
					**LYS158**	3.29

Significant values are in bold.

Figure 7 illustrates that ligand 1 and 2 engage in Pi-alkyl and alkyl interactions with amino acid residues ALA174, PRO34, PRO147, TYR145, and ALA31 within the VIH-1 (5W4Q) protein complex. On the other hand, Fig. 8 shows an alkyl interaction with amino acid residues LYZ158, CYS198, CYS218, and ILE201 within the protein 1A8O complex.

In Fig. 8, a carbon-hydrogen bond is observed with amino acid residue ASN195 in the ligand 1 when docked with the 1A8O protein, at a distance of 2.07 Å. Likewise, within the ligand 2 complex interacting with protein 1A8O, a carbon-hydrogen bond is detected with the amino acid residue ASN195, occurring at a distance of 2.04 Å.

In interaction analysis between AZT and the VIH-1 main protease 5W4Q, it is observed that OH and O form conventional hydrogen bond interactions with the ASN-139 and LYS-182 residues, respectively. Additionally, there are p-alkyl interactions with the ILE-37, PRO-34, and ARG-173 residues, as revealed by the docking results.

Conversely, in the case of protein 1A8O, conventional hydrogen bond interactions are detected at amino acid residues LYS158, PRO-157, PHE-161, GLN-155. Furthermore, there is a Pi-Sulfur interaction with CYS-218 and CYS198, as well as attractive charge interactions with amino acid GLY-220, according to the docking analysis.

Based on the visualized interactions of the ligands (P3) and (P4) with 5W4Q, we observe a significant interaction for the inhibition of HIV-1 compared to AZT, furthermore, the binding affinity infers that [5W4Q-ligandP3] and [5W4Q-ligandP4] exhibit − 5.7 kcal/mol and − 5.6 kcal/mol, respectively, representing less affinity among the studied ligands. These findings suggest their potential as drug candidates against the HIV-1 5W4Q protein (Table 3).

## Conclusion

The EtAlCl_2_ LA catalyzed DA reaction of carvone **1R** and isoprene **2**, has been studied within MEDT. The chemo-, regio-, and diastereoselectivity aspects of this LA catalyzed DA reaction have been analyzed by performing DFT calculations at B3LYP/6–311++ G(d, p) computational level. This DA reaction proceeds through an asynchronous, non-concerted molecular mechanism via a *two-stage one-step* process in which the formation of the second C1–C6 single bond begins when 80% of the first C4-C5 single bond has already been formed. Analyses of the global and local electrophilicity ω indices explain the role of the LA catalyst in the increase of the reactivity, chemo- and regioselectivity of the LA-catalyzed DA reaction. Analysis of the GEDT at the TSs provided clear evidence of substantial enhancement of the polar character of the LA catalyzed DA reaction, explaining the acceleration of the catalyzed DA reaction.

Ultimately, we conducted a docking analysis of the **P-3** and** P-4** products derived from the DFT study to assess their potential as HIV-1 inhibitors. The results indicate that **P-3** and **P-4** exhibit greater anti-HIV activity against the 5W4Q protein compared to the drug azidothymidine (AZT). These insights from the docking analysis may have implications for the design and development of new drug candidates for combating HIV-1.

### Supplementary Information

Supplementary Information.

## Data Availability

The authors confirm that the data supporting the findings of this study are available within the article and its supplementary material.

## Code availability

The calculations were performed using Gaussian 16 software provided by Gaussian, BIOVIA Discovery and Studio Visualizer provided by BIOVIA and PyMol software provided by Schrödinger.
