# Supplementary material for: MEDT analysis of mechanism and selectivities in non-catalyzed and lewis acid-catalyzed diels–alder reactions between R-carvone and isoprene
Source: Sci Rep. 2024 Jul 22;14:16827. doi: 10.1038/s41598-024-67351-9 (PMC11263594; doi:10.1038/s41598-024-67351-9)
Supplement: Supplementary file 1 — Supplementary Information. [file 41598_2024_67351_MOESM1_ESM.docx]

**MEDT Analysis of Mechanism and Selectivities in Non-Catalyzed and Lewis Acid-Catalyzed Diels-Alder Reactions between R-Carvone and Isoprene**

Khadija El Idrissi^1,2^, Mohamed Abdoul-Hakim ^1,2^, Na'il Saleh^3*^, Hocine Garmes^2^, Asad Syed^4^, Mar Ríos-Gutiérrez^5^, Bilal Ahamad Paray^6^, Meenakshi Verma^7^, Abdellah Zeroual^1*^, Luis R. Domingo^5^

*^1^ Molecular Modelling and Spectroscopy Research Team, Faculty of Science, Chouaïb Doukkali University, P.O. Box 20, 24000 El Jadida, Morocco.*

*^2^ Analytical Chemistry and Environmental Sciences Team, Department of chemistry, Faculty of Science, University Chouaib Doukkali, El Jadida*

*^3^ Department of Chemistry, College of Science, United Arab Emirates University, Al Ain P.O. Box 15551, United Arab Emirates.*

*^4^ Department of Botany and Microbiology, College of Science, King Saud University, P.O. Box 2455 Riyadh 11451, Saudi Arabia*

*^5^ Department of Organic Chemistry, University of Valencia, Dr. Moliner 50, 46100 Burjassot, Valencia, Spain*

*^6^ Department of Zoology, College of Science, King Saud University, PO Box 2455, Riyadh,11451, Saudi Arabia.*

*^7^ University Centre for Research & Development, Department of Chemistry, Chandigarh University*

*Gharuan, Mohali, India*

*^*^* [n.saleh@uaeu.ac.ae](mailto:n.saleh@uaeu.ac.ae)

*^*^* [*zeroualabdellah2@gmail.com*](mailto:zeroualabdellah2@gmail.com)

| **Table of Contents** | **Pages** |
| --- | --- |
| **Table S1.** B3LYP/6-311++G(d,p) total energies (E, in a.u.), enthalpies (H, in a.u.), entropies (S, in cal·mol-1·K-1) and Gibbs free energies (G, in a.u.), and relative energies (ΔE, in kcal·mol-1), relative enthalpies (ΔH, in kcal·mol-1), entropies (ΔS, in cal·mol-1·K-1) and Gibbs free energies (ΔG, in kcal·mol-1), computed at 25 ºC and 1atm in Toluene, for the stationary points involved in the cycloadditions [2+4] of Diels-Alder between R-Carvone 1R and Isoprene 2 in the absence of EtAlCl_2_ Lewis Acid. | **2-S** |
| **Table S2.** B3LYP/6-311++G(d,p) energy (E, in a.u.), enthalpies (H, in a.u.), entropies (S, in cal·mol^-1^·K^-^) and Gibbs free energies (G, in a.u.), and relative enthalpies (ΔH, in kcal·mol^-1^), entropies (ΔS, in cal·mol^-1^·K^-1^) and Gibbs free energies (ΔG, in kcal·mol^-1^), computed at 25 ºC and 1 atm in toluene with catalyst EtAlCl_2_ Lewis acid catalyst, for the stationary points involved in the **[4+2]**Cycloaddition reaction of R-Carvone **(1R)** and Isoprene **(2)**. | **3-S** |
| **Table S3.** ELF topological analysis of the **C_1_–C_6_** and **C_4_–C_5_** bond formation along the [4+2] Cycloaddition reaction between R-carvone **(1R)** and isoprene **(2)** in the absence of LA catalyst. Distances are given in angstroms, Å. | **4-S** |
| **Table S4**. ELF topological analysis of the **C_1_–C_6_** and **C_4_–C_5_** bond formation along the [4+2] Cycloaddition reaction between R-carvone **(1R)** and isoprene **(b)** in the presence of EtAlCl_2_ LA catalyst. Distances are given in angstroms, Å. | **5-S** |
| B3LYP/6-31G(d) Cartesian coordinates and electronic energies for all optimized structures, together with the single imaginary frequencies for TSs | **7-S** |

**Table S1.** B3LYP/6-311++G(d,p) total energies (E, in a.u.), enthalpies (H, in a.u.), entropies (S, in cal·mol^-1^·K^-1^) and Gibbs free energies (G, in a.u.), and relative energies (ΔE, in kcal·mol^-1^), relative enthalpies (ΔH, in kcal·mol^-1^), entropies (ΔS, in cal·mol^-1^·K^-1^) and Gibbs free energies (ΔG, in kcal·mol^-1^), computed at 25 ºC and 1atm in Toluene, for the stationary points involved in the cycloadditions [2+4] of Diels-Alder between R-Carvone (**1R**)and Isoprene(**2**) in the absence of EtAlCl2

Lewis Acid

| **System** | **E (a.u)** | **∆E** | **G (a.u)** | **∆G** | **H (a.u)** | **∆H** | **S** | **∆S** |
| --- | --- | --- | --- | --- | --- | --- | --- | --- |
| **1R** | -464.8293077 | - | -464,651804 | - | -464,602042 | - | 104,785 | - |
| **2** | -195,3652328 | - | -195,281739 | - | -195,245874 | - | 75,522 | - |
| **1R+2** | -660,194541 | - | -659,933543 | - | -659,847916 | - | 180,307 | - |
| **TSn-1** | -660.1494274 | 28.30 | -659.864013 | 43,63 | -659.801271 | 29.27 | 132.117 | -48.19 |
| **P-n-1** | -660.2305137 | -22.57 | -659.938253 | -2.96 | -659.877782 | -18.74 | 127.335 | -52.97 |
| **TSx-1** | -660,1499926 | 27,96 | -659,865244 | 42,86 | -659,802061 | 28.77 | 133,046 | -47,26 |
| **P-x-1** | -660,2305138 | -22,57 | -659,938251 | -2,95 | -659,877782 | -18,57 | 127,332 | -52,98 |
| **TSn-2** | -660,1502918 | 27,767 | -659,865461 | 42,72 | -659,802170 | 28,74 | 133,275 | -47,03 |
| **P-n-2** | 660,2334334 | -24,41 | -659,941048 | -4,70 | -659,880766 | 20,61 | 126,939 | -53,37 |
| **TSx-2** | -660,1482562 | 29,04 | -659,863739 | 43,80 | -659,800164 | 29,96 | 133,872 | -46,44 |
| **P-x-2** | -660,2334334 | -24,41 | -659,941051 | -4,71 | -659,880765 | -20,61 | 126,946 | -53,36 |
| **TSn-3** | -660,1500727 | 27,90 | -659,865362 | 42,78 | -659,801897 | 28,88 | 133,640 | 46,67 |
| **P-n-3** | -660,2311595 | -22,98 | -659,938930 | -3,38 | -659,878389 | -19,12 | 127,482 | -52,83 |
| **TSx-3** | -660,1490372 | 28.55 | -659,864060 | 43,60 | -659,801019 | 29,43 | 132,748 | 47,56 |
| **P-x-3** | -660,2333244 | -24,34 | -659,941063 | -4,72 | -659,880657 | -20,55 | 127,199 | -53,11 |

**Table S2.** B3LYP/6-311++G(d, p) energy (E, in a.u.), enthalpies (H, in a.u.), entropies (S, in cal·mol^-1^·K^-^) and Gibbs free energies (G, in a.u.), and relative enthalpies (ΔH, in kcal·mol^-1^), entropies (ΔS, in cal·mol^-1^·K^-1^) and Gibbs free energies (ΔG, in kcal·mol^-1^), computed at 25 ºC and 1 atm in toluene with catalyst EtAlCl_2_ Lewis acid catalyst, for the stationary points involved in the [4+2]Cycloaddition reaction of R-Carvone **(1R)** and Isoprene **(2)**.

| System | E | ∆E | G (a.u) | ∆G | H (a.u) | ∆H | S | ∆S |
| --- | --- | --- | --- | --- | --- | --- | --- | --- |
| 1R-Al | -1707,1846621 | - | -1706,952774 | - | -1706.878935 | - | 155.484 | - |
| 2 | -195,3652328 | - | -195,281739 |  | -195.245874 | - | 75.522 | - |
| 1R-Al +2 | -1902,549895 | - | -1902,234513 | - | -1902,124809 | - | 231,006 | - |
| TSn-1-Al | -1902,5206659 | 18,34 | -1902,180814 | 33,70 | -1902.093680 | 19,53 | 183.481 | -47,53 |
| P-n-1-Al | -1902,582582 | -20,51 | -1902.235685 | -0,74 | -1902.151227 | -16,58 | 177.848 | -53,16 |
| TSx-1-Al | -1902.5082454 | 26,14 | -1902.167569 | 42,01 | -1902.081218 | 27,35 | 181.834 | -49,17 |
| P-x-1-Al | -1902,5826816 | -20.57 | -1902,235862 | -0.85 | -1902,151226 | -16,57 | 177,798 | -53,21 |
| TSx-2-Al | -1902,5161768 | 21,16 | -1902,178863 | 34,92 | -1902,089382 | 22,23 | 188,423 | 34,92 |
| P-x-2-Al | -1902,5837614 | -21,25 | -1902,237828 | -2.08 | -1902,152511 | -17,38 | 179.656 | -51,35 |
| TSn-2-Al | -1902,5062071 | 27,41 | -1902.166950 | 42,40 | -1902.078980 | 28,76 | 185.242 | -45,76 |
| P-n-2-Al | -1902,5870296 | -23,30 | -1902.239835 | -3,34 | -1902,148188 | -14,67 | 177,191 | -53,82 |
| TSn-3-Al | -1902,5171163 | 20,57 | -1902,177937 | 35,50 | -1902,090140 | 21,76 | 184,878 | -46,13 |
| P-n-3-Al | -1902,5815888 | -19,89 | -1902,235744 | -0,77 | -1902,150281 | -15.98 | 179,962 | -51,04 |
| TSx-3Al | -1902,5177335 | 20,18 | -1902,178188 | 35,34 | -1902,090912 | 21,27 | 183,779 | -47,23 |
| P-x-3-Al | -1902,5836519 | -21,18 | -1902,237402 | -1,81 | -1902,152377 | -17.30 | 179,042 | -51.96 |

**Table S3.** ELF topological analysis of the **C_1_–C_6_** and **C_4_–C_5_** bond formation along the [4+2] Cycloaddition reaction between R-carvone **(1R)** and isoprene **(2)** in the absence of EtAlCl_2_ LA catalyst. Distances are given in angstroms, Å.

|  | **d(C1,C6)** | **d(C4,C5)** | **V(C1,C2)** | **V’(C1,C2)** | **V(C1)** | **V(C2)** | **V(C1,C6)** | **V(C4,C5)** | **V(C4)** | **V(C5)** | **V(C6)** | **V(C2,C3)** | **V’(C2,C3)** | **V(C3,C4)** | **V’(C3,C4)** | **V(C5,C6)** | **V’(C5,C6)** |
| --- | --- | --- | --- | --- | --- | --- | --- | --- | --- | --- | --- | --- | --- | --- | --- | --- | --- |
| **S1** | 3.36 | 3.07 | 1.65 | 1.67 | - | - | - | - | - | - | - | 2.22 | - | 1.67 | 1.69 | 1.69 | 1.66 |
| **S2** | 2.81 | 2.32 | 1.59 | 1.65 | - | - | - | - | - | - | - | 2.35 | - | 3.22 | - | 3.22 | - |
| **S3** | 2.79 | 2.27 | 3.22 | - | - | - | - | - | - | - | - | 2.38 | - | 3.19 | - | 3.20 | - |
| **S4** | 2.74 | 2.17 | 3.18 | - | - | - | - | - | - | - | - | 2.44 | - | 3.15 | - | 3.16 | - |
| **S5** | 2.72 | 2.12 | 3.15 | - | - | - | - | - | - | - | - | 2.49 | - | 3.14 | - | 3.14 | - |
| **S6** | 2.70 | 2.07 | 3.12 | - | - | - | - | - | - | 0.19 | - | 2.53 | - | 3.14 | - | 2.93 | - |
| **TSn-1** | 2.65 | 2.00 | 3.04 | - | - | - | - | - | 0.47 | 0.37 | - | 2.65 | - | 2.64 | - | 2.68 | - |
| **S8** | 2.60 | 1.88 | 2.95 | - | - | - | - | 1.12 | - | - | - | 2.79 | - | 2.46 | - | 2.50 | - |
| **S9** | 2.57 | 1.83 | 2.91 | - | - | - | - | 1.23 | - | - | - | 2.86 | - | 2.40 | - | 2.42 | - |
| **S10** | 2.54 | 1.79 | 2.86 | - | - | - | - | 1.33 | - | - | - | 2.92 | - | 2.34 | - | 2.36 | - |
| **S11** | 2.51 | 1.75 | 2.83 | - | - | - | - | 1.42 |  |  | - | 3.01 | - | 2.27 | - | 2.30 | - |
| **S12** | 2.33 | 1.64 | 2.43 | - | 0.33 | - | - | 1.63 | - | - | 0.45 | 3.20 | - | 2.16 | - | 2.16 | - |
| **S13** | 2.28 | 1.63 | 2.38 | - | 0.38 | - | - | 1.66 | - | - | 0.50 | 3.24 | - | 2.14 | - | 2.13 | - |
| **S14** | 2.23 | 1.61 | 2.33 | - | 0.42 | - | - | 1.68 | - | - | 0.54 | 3.29 | - | 2.12 | - | 2.11 | - |
| **S15** | 2.18 | 1.61 | 2.29 | - | 0.47 | - | - | 1.70 | - | - | 0.59 | 3.32 | - | 2.10 | - | 2.09 | - |
| **S16** | 2.13 | 1.60 | 2.25 | - | 0.50 | - | - | 1.72 | - | - | 0.64 | 1.84 | 1.50 | 2.09 | - | 2.07 | - |
| **S17** | 2.07 | 1.59 | 2.21 | - | - | - | 1.21 | 1.74 | - | - | - | 1.83 | 1.54 | 2.08 | - | 2.06 | - |
| **S18** | 2.02 | 1.59 | 2.18 | - | - | - | 1.28 | 1.75 | - | - | - | 1.83 | 1.56 | 2.07 | - | 2.04 | - |
| **S19** | 1.97 | 1.58 | 2.15 | - | - | - | 1.35 | 1.76 | - | - | - | 1.82 | 1.58 | 2.06 | - | 2.02 | - |
| **S20** | 1.92 | 1.57 | 2.13 | - | - | - | 1.42 | 1.78 | - | - | - | 1.82 | 1.61 | 2.05 | - | 2.01 | - |
| **S21** | 1.87 | 1.57 | 2.10 | - | - | - | 1.48 | 1.78 | - | - | - | 1.82 | 1.63 | 2.04 | - | 2.00 | - |
| **S22** | 1.82 | 1.57 | 2.09 | - | - | - | 1.54 | 1.79 | - | - | - | 1.81 | 1.64 | 2.04 | - | 1.98 | - |
| **S23** | 1.77 | 1.56 | 2.07 | - | - | - | 1.60 | 1.80 | - | - | - | 1.81 | 1.66 | 2.03 | - | 1.97 | - |
| **S24** | 1.72 | 1.56 | 2.05 | - | - | - | 1.65 | 1.81 | - | - | - | 1.80 | 1.67 | 2.03 | - | 1.96 | - |
| **S25** | 1.68 | 1.56 | 2.04 |  |  |  | 1.70 | 1.82 | - | - | - | 1.79 | 1.69 | 2.02 | - | 1.94 | - |
| **S26** | 1.64 | 1.55 | 2.03 | - | - | - | 1.73 | 1.83 | - | - | - | 1.80 | 1.68 | 2.02 | - | 1.93 | - |
| **S27** | 1.59 | 1.55 | 2.03 | - | - | - | 1.78 | 1.85 | - | - | - | 1.80 | 1.67 | 2.02 | - | 1.93 | - |

**Table S4.** ELF topological analysis of the **C_1_–C_6_** and **C_4_–C_5_** bond formation along the [4+2] Cycloaddition reaction between R-carvone **(1R)** and isoprene **(2)** in the presence of EtAlCl_2_ LA catalyst. Distances are given in angstroms, Å.

|  | d(C1,C6) | d(C4,C5) | V(C1,C2) | V’(C1,C2) | V(C1) | V(C2) | **V(C1,C6)** | **V(C4,C5)** | V(C4) | V(C5) | V(C6) | V(C2,C3) | V’(C2,C3) | V(C3,C4) | V’(C3,C4) |  | V(C5,C6) |
| --- | --- | --- | --- | --- | --- | --- | --- | --- | --- | --- | --- | --- | --- | --- | --- | --- | --- |
| **S’1** | 4.20 | 3.87 | 1.60 | 1.74 | - | - | - | - | - | - | - | 2.20 | - | 1.76 | 1.65 |  | 3.25 |
| **S’2** | 3.52 | 3.17 | 1.57 | 1.74 | - | - | - | - | - | - | - | 2.22 | - | 1.71 | 1.65 |  | 3.22 |
| **S’3** | 3.08 | 2.44 | 1.57 | 1.66 | - | - | - | - | - | - | - | 2.30 | - | 3.23 | - |  | 3.10 |
| **S’4** | 3.07 | 2.40 | 1.57 | 1.67 | - | - | - | - | - | - | - | 2.31 | - | 3.21 | - |  | 3.09 |
| **S’5** | 3.05 | 2.35 | 3.22 | - | - | - | - | - | - | - | - | 2.33 | - | 3.19 | - |  | 3.07 |
| **S’6** | 3.03 | 2.30 | 3.22 | - | - | - | - | - | - | - | - | 2.34 | - | 3.17 | - |  | 3.05 |
| **S’7** | 3.01 | 2.24 | 3.20 | - | - | - | - | - | - | - | - | 2.36 | - | 3.15 | - |  | 3.01 |
| **S’8** | 2.30 | 2.19 | 3.18 | - | - | - | - | - | - | - | - | 2.38 | -- | 3.13 | - |  | 2.99 |
| **S’9** | 2.98 | 2.14 | 3.15 | - | - | - | - | - | - | - | - | 2.41 | - | 3.12 | - |  | 2.96 |
| **S’10** | 2.96 | 2.08 | 3.13 | - | - | - | - | - | - | 0.14 | - | 2.44 | - | 3.11 | - |  | 2.80 |
| **S’11** | 2.95 | 2.03 | 3.10 | - | - | - | - | - | 0.38 | 0.23 | - | 2.47 | - | 2.73 | - |  | 2.68 |
| **TSn-1-Al** | 2.93 | 1.97 | 3.07 | - | - | - | - | 0.79 | - | - | - | 2.50 | - | 2.63 | - |  | 2.59 |
| **S’13** | 2.92 | 1.92 | 3.04 | - | - | - | - | 0.95 | - | - | - | 2.54 | - | 2.55 | - |  | 2.50 |
| **S’14** | 2.90 | 1.86 | 3.01 | - | - | - | - | 1.08 | - | - | - | 2.58 | - | 2.47 | - |  | 2.42 |
| **S’15** | 2.88 | 1.81 | 2.97 | - | - | - | - | 1.21 | - | - | - | 2.62 | - | 2.41 | - |  | 2.36 |
| **S’16** | 2.86 | 1.76 | 2.93 | - | - | - | - | 1.32 | - | - | - | 2.67 | - | 2.35 | - |  | 2.31 |
| **S’17** | 2.83 | 1.71 | 2.89 | - | - | - | - | 1.41 | - | - | - | 2.71 | - | 2.30 | - |  | 2.27 |
| **S’18** | 2.79 | 1.69 | 2.86 | - | - | - | - | 1.48 | - | - | - | 2.75 | - | 2.27 | - |  | 2.25 |
| **S’19** | 2.75 | 1.67 | 2.83 | - | - | - | - | 1.51 | - | - | - | 2.79 | - | 2.25 | - |  | 2.23 |
| **S’20** | 2.57 | 1.63 | 2.73 |  | - | - | - | 1.59 | - | - | - | 2.92 | - | 2.20 | - |  | 2.20 |
| **S’21** | 2.53 | 1.63 | 2.70 | - | - | - | - | 1.61 | - | - | - | 2.96 | - | 2.19 | - |  | 2.19 |
| **S’22** | 2.29 | 1.60 | 2.44 | - | 0.19 | - | - | 1.70 | - | - | 0.36 | 3.18 | - | 2.12 | - |  | 2.13 |
| **S’23** | 2.08 | 1.58 | 2.25 | - | - | - | 0.97 | 1.75 | - | - | - | 1.43 | 1.88 | 2.09 | - |  | 2.06 |
| **S’24** | 2.02 | 1.57 | 2.22 | - | - | - | 1.06 | 1.76 | - | - | - | 3.34 | - | 2.07 | - |  | 2.04 |
| **S’25** | 1.97 | 1.57 | 2.19 | - | - | - | 1.15 | 1.77 | - | - | - | 1.53 | 1.85 | 2.06 | - |  | 2.02 |
| **S’26** | 1.92 | 1.57 | 2.16 | - | - | - | 1.24 | 1.78 | - | - | - | 1.56 | 1.84 | 2.06 | - |  | 2.00 |
| **S’27** | 1.86 | 1.56 | 2.12 | - | - | - | 1.33 | 1.79 | - | - | - | 1.59 | 1.84 | 2.05 | - |  | 1.99 |
| **S’28** | 1.81 | 1.56 | 2.10 | - | - | - | 1.41 | 1.80 | - | - | - | 1.61 | 1.84 | 2.04 | - |  | 1.97 |
| **S’29** | 1.76 | 1.56 | 2.07 | - | - | - | 1.49 | 1.81 | - | - | - | 1.62 | 1.84 | 2.04 | - |  | 1.96 |
| **S’30** | 1.71 | 1.55 | 2.06 | - | - | - | 1.56 | 1.82 | - | - | - | 1.64 | 1.83 | 2.03 | - |  | 1.95 |
| **S’31** | 1.67 | 1.55 | 2.04 | - | - | - | 1.62 | 1.83 | - | - | - | 1.65 | 1.83 | 2.03 | - |  | 1.94 |
| **S’32** | 1.64 | 1.55 | 2.03 | - | - | - | 1.66 | 1.83 | - | - | - | 1.65 | 1.83 | 2.02 | - |  | 1.93 |
| **S’33** | 1.63 | 1.55 | 2.03 | - | - | - | 1.67 | 1.83 | - | - | - | 1.66 | 1.83 | 2.02 | - |  | 1.93 |
| **S’34** | 1.62 | 1.55 | 2.03 | - | - | - | 1.68 | 1.83 | - | - | - | 1.66 | 1.82 | 2.02 | - |  | 1.93 |
| **S’35** | 1.61 | 1.55 | 2.02 | - | - | - | 1.70 | 1.84 | - | - | - | 1.66 | 1.82 | 2.02 | - |  | 1.93 |
| **S’36** | 1.60 | 1.55 | 2.02 | - | - | - | 1.72 | 1.86 | - | - | - | 1.70 | 1.81 | 2.03 | - |  | 1.92 |
| **S’37** | 1.60 | 1.55 | 2.02 | - | - | - | 1.72 | 1.85 | - | - | - | 1.70 | 1.79 | 2.03 | - |  | 1.93 |
| **S’38** | 1.60 | 1.55 | 2.02 | - | - | - | 1.73 | 1.87 | - | - | - | 1.68 | 1.81 | 2.03 | - |  | 1.92 |
| **S’39** | 1.60 | 1.55 | 2.02 | - |  |  | 1.73 | 1.85 |  |  |  | 1.69 | 1.79 | 2.03 | - |  | 1.92 |
| **S’40** | 1.60 | 1.55 | 2.02 | - | - | - | 1.73 | 1.85 | - | - | - | 1.69 | 1.80 | 2.03 | - |  | 1.92 |

B3LYP/6-311++G(d,p) Cartesian coordinates and electronic energies for TSs structures, together with the single imaginary frequencies for the [4+2] cycloadditions of Diels-Alder between R-Carvone **(1R)** and Isoprene **(2)** in the absence of **EtAlCl_2_** Lewis Acid.

**TSn-1**

E(RB3LYP) = -660.149427A.U.

1 imaginary frequency -462.87 cm^-1^

C 2.17985000 0.61623900 1.49653300

C 1.21461700 1.58694100 1.45714600

C 0.82037800 2.25576900 0.27672300

C 1.35904500 1.85834800 -0.96077900

C 0.78217200 0.01303100 -1.36969400

C 1.53392200 -0.89455000 -0.58234800

C -0.30199500 3.26155600 0.33081000

C -0.73914200 -0.04887600 -1.33823600

C -1.29955500 -0.44707300 0.04368300

C -0.63620800 -1.74904300 0.50049200

C 0.87955200 -1.81782500 0.34068500

O 1.49048900 -2.73721700 0.89452400

C 2.95728300 -1.20168500 -0.97213500

C -2.82339900 -0.47918100 0.05041500

C -3.50959900 0.85861800 -0.10288200

C -3.54240900 -1.59549900 0.20173100

H 0.61022300 1.74744600 2.34694600

H 2.41367300 1.60865000 -1.00458400

H 1.01963700 2.40226600 -1.83841200

H -0.97856600 3.08031300 1.16946600

H 0.10548800 4.27199000 0.45802800

H -0.88449200 3.26988900 -0.59318900

H -1.15812400 0.90294300 -1.67029200

H -1.06062400 -0.79876100 -2.07378700

H -1.00692800 0.34011400 0.74705800

H -1.01342300 -2.59257600 -0.09170100

H -0.87635000 -1.97670800 1.54198500

H 2.98159200 -1.93576100 -1.78702400

H 3.47910400 -0.30984900 -1.33462400

H 3.51620400 -1.62850300 -0.13926500

H -3.27009800 1.33323400 -1.05976100

H -4.59447700 0.75494200 -0.04409600

H -3.18963000 1.55243500 0.68220800

H -4.62695500 -1.56138800 0.20354000

H -3.09362700 -2.57198000 0.33319500

H 1.17168500 0.13885600 -2.37904700

H 2.96159600 0.55329000 0.75695800

H 2.32310000 0.01628400 2.38734000

**TSx-1**

E(RB3LYP) = -660.149993 A.U.

1 imaginary frequency -463.88 cm^-1^

C -1.65741700 0.42523500 1.79276400

C -2.72867200 -0.24992700 1.26890600

C -2.60747300 -1.29725500 0.32749600

C -1.32921800 -1.67696000 -0.11150700

C -0.44308700 -0.19821600 -1.13289300

C -0.76986300 1.07559300 -0.60538700

C 1.01067500 -0.65194100 -1.13998400

C 1.77230500 -0.18296300 0.11711200

C 1.65243900 1.33955400 0.22535600

C 0.24953700 1.91052800 0.02808700

O 0.05147200 3.09361600 0.31587800

C -2.04209500 1.72988600 -1.06486800

C 3.19731500 -0.72070800 0.15509600

C 3.32369700 -2.19927200 0.43885300

C 4.28223900 0.03711600 -0.02746100

H -1.26897000 -2.48584800 -0.83413300

H 1.06421800 -1.73753900 -1.24949900

H 1.50264600 -0.22837600 -2.02518700

H 1.26820600 -0.62272700 0.98862500

H 2.25896700 1.81883000 -0.55316900

H 2.03566100 1.70753600 1.18052600

H -1.94798500 2.05715300 -2.10855200

H -2.87544600 1.02125000 -1.02862900

H -2.28635400 2.60184100 -0.45947100

H 2.81230400 -2.80460100 -0.31644900

H 4.36932600 -2.51060000 0.46614600

H 2.86737400 -2.44832800 1.40379100

H 5.27587800 -0.39721500 0.00810300

H 4.23085000 1.10288500 -0.21206700

H -0.97801700 -0.44896100 -2.04668700

H -1.80590500 1.32621800 2.37580900

H -3.72363500 0.14944600 1.44732100

C -3.83508500 -1.85722300 -0.34613500

H -3.70133800 -1.90934800 -1.43158300

H -4.03493300 -2.87900100 -0.00397900

H -4.72354800 -1.25771700 -0.13783700

H -0.66747900 -0.00203800 1.83227900

H -0.53538300 -1.70248000 0.62501300

**TSn-2**

E(RB3LYP) = -660.150292A.U.

1 imaginary frequency -476.24cm^-1^

C -2.08618900 -1.22095300 1.16517700

C -1.90467900 -1.85747600 -0.07135800

C -2.02075900 -1.10610200 -1.25974800

C -2.43169300 0.20403600 -1.29168900

C -1.00904000 1.29747400 0.51319900

C -0.61719600 0.18040000 1.29003800

C 0.70608000 -0.52279200 1.00433000

C 1.74127700 0.41889200 0.35321600

C 1.09557000 1.01785200 -0.91329200

C -0.15614100 1.81268300 -0.56492300

O -0.37283500 2.89614100 -1.11093700

C 3.07212500 -0.26233000 0.08234200

C 4.18170400 0.15263600 0.70159300

H -1.61596100 -1.54251800 -2.16982700

C 3.10722500 -1.40775100 -0.90066600

C -2.05592600 2.23775100 1.05757800

H -0.85281800 0.27803400 2.34710500

H 1.10425700 -0.94130700 1.93287500

H 0.54725000 -1.36120800 0.32271200

H 1.93905300 1.24319500 1.04737100

H 1.77716900 1.69052200 -1.43752500

H 0.82073400 0.21472600 -1.60675500

H -2.34951100 0.78463500 -2.20278600

H 5.14302200 -0.31858300 0.52381500

H 4.16458800 0.97949800 1.40438600

H 2.45063100 -2.22705700 -0.59060800

H 2.77135400 -1.09252500 -1.89390100

H 4.11842500 -1.80660000 -0.99842200

H -1.93988300 -1.81721200 2.06170900

H -1.60662400 2.94048400 1.76942400

H -2.50841100 2.83243900 0.26375700

H -2.84286300 1.69873400 1.59383700

C -1.36720400 -3.26637800 -0.12989200

H -0.80102900 -3.51959200 0.76972300

H -2.19298500 -3.98466600 -0.19877500

H -0.72751700 -3.42615000 -1.00199900

H -3.07981400 0.61598600 -0.53381400

H -2.86731200 -0.47763000 1.26976800

**TSx-2**

E(RB3LYP) = -660.148256A.U.

1 imaginary frequency -476.16cm^-1^

C 0.67810500 0.89688300 -0.58279800

C 0.40660700 -0.46028400 -0.89225000

C -0.97177500 -1.06965100 -0.61482700

C -2.06946800 -0.00422300 -0.41045000

C -1.56365700 1.02106100 0.62504900

C -0.31655700 1.72599800 0.10701300

C 1.94384600 0.69949400 1.61576800

O -0.20954300 2.94856500 0.20712100

C -3.41502500 -0.61165500 -0.05019700

C -4.43941800 -0.53893500 -0.90577500

C -3.56199000 -1.28798500 1.29157800

C 3.02016500 0.06306400 1.04642800

C 2.90453400 -1.13260700 0.30603800

C 1.63337200 -1.70477100 0.15513700

C 4.07889500 -1.66281600 -0.47814900

C 1.76463200 1.59425700 -1.35583900

H 0.84296900 -0.78147900 -1.83407000

H -1.24421300 -1.72434900 -1.44732900

H -0.94359100 -1.70719700 0.27400300

H -2.20132700 0.52758800 -1.35934900

H -2.31296600 1.78602500 0.83727300

H -1.32986700 0.51670800 1.57109700

H 2.04993700 1.69537400 2.02895000

H 1.04735200 0.16795600 1.89984300

H -5.40770100 -0.96975500 -0.67225800

H -4.34302900 -0.04356100 -1.86661000

H -2.83751400 -2.09850700 1.42084000

H -3.39606300 -0.58285900 2.11265400

H -4.56185600 -1.70964500 1.40800900

H 3.96786700 0.59329600 1.00343200

H 1.54867300 -2.62108000 -0.42236600

H 0.96785800 -1.66927600 1.00761000

H 4.44089100 -2.60472600 -0.05138000

H 3.79789400 -1.87416700 -1.51523500

H 4.91327000 -0.95852700 -0.48656900

H 1.43881100 1.78121100 -2.38704500

H 2.02471600 2.55216800 -0.90678000

H 2.66064000 0.96929800 -1.41203800

**TSn-3**

E(RB3LYP) = -660.150073A.U.

1 imaginary frequency - -469.37 cm^-1^

C -1.57989200 1.87526800 -0.35458300

C -1.17950600 1.93189100 0.99727800

C -1.49834900 0.93541300 1.93250600

C -0.61021600 -0.73697600 1.42187400

C -1.24915300 -1.28100300 0.27884700

C -2.38223500 0.84446000 -0.78675800

C -0.99330900 2.86042300 -1.34366500

C 0.89123500 -0.48887900 1.38990900

C 1.38454600 -0.01649200 0.01169900

C 0.92773600 -1.00708300 -1.07440400

C -0.51911000 -1.48483500 -0.97349600

O -0.99109000 -2.13904600 -1.90675600

C 2.88567500 0.23285700 -0.02700800

C 3.36606000 1.43513700 -0.35939800

C 3.81303000 -0.90779300 0.31822400

C -2.54168200 -2.03749100 0.45074400

H -0.41753700 2.65902400 1.26552900

H -1.12624800 1.07996900 2.94252200

H -2.49609300 0.50805100 1.91091400

H -0.94014400 -1.19047000 2.35586000

H -3.03518100 0.31240700 -0.11503100

H -2.56061200 0.68466500 -1.84408600

H 0.06160900 3.06533200 -1.14286600

H -1.08396700 2.49452100 -2.36815200

H -1.52453100 3.81626500 -1.28839600

H 1.17511900 0.23727800 2.15737700

H 1.38479100 -1.43171100 1.65716200

H 0.90368800 0.94169900 -0.19254100

H 1.52938900 -1.92360500 -1.03894900

H 1.07609400 -0.58727100 -2.07285200

H 4.43279000 1.63064300 -0.40030600

H 2.70857300 2.26284400 -0.60494900

H 3.66965600 -1.24164300 1.35109200

H 3.64036400 -1.77934700 -0.32096700

H 4.85658800 -0.60931300 0.20438500

H -2.34022200 -3.06915800 0.76461200

H -3.17078700 -1.58665500 1.22477200

H -3.10360900 -2.09055800 -0.48169900

**TSx-3**

E(RB3LYP) = -660.149037A.U.

1 imaginary frequency -483.81cm^-1^

C 1.79352500 -0.10945000 -1.43480400

C 2.74210600 -0.85915500 -0.77359700

C 2.33920600 -1.77701600 0.21852700

C 0.99645800 -1.95459000 0.57875400

C 0.21855700 -0.28757300 1.34689600

C 0.79829800 0.86146200 0.75220500

C -1.27939000 -0.52609100 1.20862500

C -1.81504600 -0.08357400 -0.16902800

C -1.44963400 1.38688400 -0.39074400

C -0.00647300 1.76948600 -0.07024600

O 0.40241400 2.87645900 -0.42373200

C 2.09277700 1.36950500 1.31709400

C -3.29338300 -0.41045800 -0.33921700

C -3.61742800 -1.87746700 -0.49895900

C -4.25954500 0.51167200 -0.36886700

H 0.78456200 -2.66927700 1.36787300

H -1.50893700 -1.57714900 1.39935000

H -1.79844200 0.04835900 1.98678400

H -1.29315100 -0.67905700 -0.93042300

H -2.05629700 2.02517900 0.26369400

H -1.66445700 1.70693700 -1.41348800

H 1.92746500 1.81510200 2.30634100

H 2.79787800 0.54295400 1.45847100

H 2.54394600 2.12613700 0.67671600

H -3.30143600 -2.46192100 0.37104000

H -4.68849600 -2.03461500 -0.63709600

H -3.09534100 -2.29695100 -1.36658900

H -5.29840600 0.22401100 -0.49213500

H -4.06332200 1.57273600 -0.27818900

H 0.59951400 -0.50643100 2.34174600

H 2.08726100 0.71505200 -2.07437000

H 0.77382300 -0.44385100 -1.54004600

H 0.26206200 -1.97396700 -0.21875800

C 4.21647200 -0.57960700 -0.97380900

H 4.37589100 0.40246800 -1.42323000

H 4.77030100 -0.62154700 -0.03235800

H 4.65807300 -1.32284100 -1.64600500

H 3.10921000 -2.20716000 0.85354400

B3LYP/6-311++G(d,p) Cartesian coordinates and electronic energies for TSs structures, together with the single imaginary frequencies for the cycloadditions [2+4] of Diels-Alder between R-Carvone **(1R)** and Isoprene **(2)** in the presence of **EtAlCl_2_** Lewis Acid.

**TSn-1-Al**

E(RB3LYP) = -1902.5206659A.U.

1 imaginary frequency -375.91 cm^-1^

C 0.92753600 -2.26931500 1.46027500

C 2.13372800 -1.70353100 1.72259900

C 3.24012900 -1.68523500 0.82177500

C 3.13136700 -2.18955100 -0.48318100

C 2.00384300 -0.99567400 -1.57779500

C 0.64180800 -1.28824800 -1.28960300

C 4.50380400 -1.00036300 1.26159200

C 2.49483500 0.43649800 -1.46873100

C 1.86040300 1.20608200 -0.29333600

C 0.33597300 1.05457700 -0.32772200

C -0.17127800 -0.32456100 -0.66309800

O -1.42887700 -0.56096400 -0.51397600

C 0.01947100 -2.55555700 -1.81692400

C 2.33720300 2.65291900 -0.22708200

C 3.82335200 2.84772600 -0.03128400

C 1.51915900 3.70645100 -0.29978100

H 2.24044500 -1.13099900 2.63994200

H 2.50482300 -3.05908300 -0.64315600

H 4.04425700 -2.20442500 -1.07057200

H 4.30755700 -0.14950300 1.91760600

H 5.11190400 -1.71039000 1.83652300

H 5.10841000 -0.67185200 0.41508200

H 3.58402200 0.45508900 -1.40862500

H 2.23156900 0.93921600 -2.40920400

H 2.21374200 0.73107500 0.62867200

H -0.10142200 1.70633100 -1.09497700

H -0.10699100 1.37371300 0.61863000

H -0.36021400 -2.40039400 -2.83337900

H 0.74964600 -3.36824900 -1.87370300

H -0.82321600 -2.87882300 -1.20617100

H 4.40022800 2.48804500 -0.88938500

H 4.06341400 3.90251200 0.11047300

H 4.18073000 2.29716800 0.84573900

H 1.91151400 4.71533600 -0.22864100

H 0.44690300 3.62030400 -0.42020800

H 2.37580200 -1.50062100 -2.46729200

Al -2.90523600 0.08817300 0.36933000

C -4.42892700 -1.10195000 0.02466000

C -4.87217100 -1.22835700 -1.44705900

H -4.06880500 -1.62263700 -2.07831200

H -5.73209000 -1.89832000 -1.56425200

H -5.15970100 -0.25806700 -1.86390400

H -4.18417300 -2.09541600 0.42448700

Cl -2.24514500 0.12063700 2.46618100

Cl -3.18600300 2.14497200 -0.32932600

H -5.27268200 -0.74815300 0.63172100

H 0.76946800 -2.95358200 0.64021700

H 0.09205500 -2.12363000 2.13484200

**TSx-1-Al**

E(RB3LYP) = -1902.508245A.U.

1 imaginary frequency -385.20 cm^-1^

C 1.32711000 -1.63127100 1.73638600

C 2.26591100 -2.50678600 1.28631500

C 3.40030400 -2.14221300 0.50533300

C 3.59617200 -0.80663700 0.11579600

C 2.30830000 -0.22606000 -1.25678800

C 1.03976200 -0.84096700 -1.07437600

C 2.40309200 1.29037300 -1.17756100

C 1.51985900 1.87309500 -0.05656500

C 0.08346900 1.36462900 -0.21398300

C -0.05389800 -0.09440700 -0.57233100

O -1.21638000 -0.62259200 -0.53586300

C 0.83567100 -2.23411900 -1.58749200

C 1.63908400 3.38882300 0.05073100

C 3.01543200 3.91220900 0.39193400

C 0.60812100 4.22279400 -0.10135600

H 4.52848000 -0.57903800 -0.39260500

H 3.44453400 1.59526700 -1.05586200

H 2.07207700 1.69854000 -2.14131400

H 1.90667900 1.48485100 0.89691100

H -0.43142500 1.90189800 -1.02051600

H -0.50438700 1.56165100 0.68582100

H 0.83034700 -2.24473700 -2.68402700

H 1.66138700 -2.88263500 -1.27194900

H -0.10267500 -2.66025900 -1.23759000

H 3.73552200 3.73388600 -0.41359200

H 2.98989400 4.98671500 0.57847400

H 3.41230500 3.42288300 1.28919100

H 0.74454900 5.29336700 0.00670000

H -0.39887100 3.89166900 -0.32045000

H 2.88422900 -0.65510500 -2.07462700

Al -2.87132600 -0.31508800 0.27149500

C -4.06016800 -1.80413800 -0.20003200

C -4.36824500 -1.97303300 -1.70140100

H -3.45812000 -2.15669300 -2.28262600

H -5.04711200 -2.81247100 -1.89241700

H -4.83713200 -1.07594000 -2.11691700

H -3.62591800 -2.73128700 0.19787700

Cl -2.27648900 -0.22593800 2.37720000

Cl -3.49293100 1.64614400 -0.43928700

H -4.99898500 -1.66902800 0.35273700

H 0.41509500 -1.97245400 2.21096900

H 2.08713000 -3.56882600 1.43030900

C 4.32464600 -3.21659300 0.00020700

H 4.70929300 -2.97977700 -0.99534200

H 5.19425600 -3.30124000 0.66360500

H 3.84321600 -4.19580400 -0.03140300

H 1.47916600 -0.56213100 1.73896300

H 3.29713500 -0.04412700 0.82372300

**TSn-2-Al**

E(RB3LYP) = -1902.506207A.U.

1 imaginary frequency -392.75cm^-1^

C -2.84628800 0.44452600 -0.92952800

C -1.96485400 1.68060700 -0.65320800

C -0.71186700 1.21491300 0.11656800

C 0.05356600 0.19076300 -0.68671000

O 1.32899200 0.23635500 -0.70494900

C -2.72127000 2.79285500 0.05332500

C -2.97197800 3.94123600 -0.58082000

H -1.53495900 -1.98896300 2.29765800

C -3.16728200 2.56866500 1.47793000

C 0.14109300 -1.54100600 -2.51911700

H -2.45264000 -0.97723400 -2.56868800

H -3.68908200 0.72281500 -1.56805800

H -3.27236600 0.11708900 0.01969600

H -1.63070900 2.07971800 -1.61669100

H -0.04283800 2.04862600 0.33366100

H -0.98857200 0.77727400 1.08139900

H 0.65395700 -2.23178400 1.27094900

H -3.51310600 4.74976900 -0.10110900

H -2.63918000 4.11420900 -1.59900800

H -3.80545700 1.68365800 1.57231600

H -2.31243200 2.41764900 2.14510000

H -3.73160800 3.42608700 1.84773400

H -3.68275600 -2.51578400 -1.34663300

H 0.27318000 -1.00149900 -3.46422200

H 1.13518000 -1.77726100 -2.14025100

H -0.37965500 -2.47351200 -2.75900400

Al 2.81951800 0.27974300 0.40483300

Cl 3.54966400 -1.76352900 0.19328600

Cl 1.93592100 0.50321500 2.39548200

C 4.04625000 1.70465500 -0.15709200

H 3.55175400 2.67167200 0.00710500

H 4.89870900 1.70020800 0.53462800

C 4.56123700 1.62112000 -1.60824200

H 5.09217000 0.68196300 -1.79104400

H 3.74061800 1.67146800 -2.33186400

H 5.25265300 2.43721200 -1.84824000

C -3.98835900 -2.14403800 1.28281200

H -4.80549100 -1.83753500 0.62750500

H -4.28481100 -3.09254400 1.74782900

H -3.88667300 -1.41573200 2.09212700

H -0.09399700 -2.84596000 -0.28610800

H -1.94048300 -3.01004400 -1.35978700

**TSx-2-Al**

E(RB3LYP) = -1902,5161768A.U.

1 imaginary frequency -359.60cm^-1^

C 1.15712500 -0.52171800 1.11491800

C 2.46928400 0.03000500 0.99161200

C 2.64940300 1.50254000 0.59182700

C 1.31403800 2.26643100 0.46341100

C 0.28659000 1.38609800 -0.27841400

C 0.06769200 0.09736800 0.47823000

C 1.11663300 -2.25275800 -1.41996100

O -1.12332500 -0.36936900 0.61133500

C 1.49461000 3.63782300 -0.16609300

C 1.36076200 4.73975800 0.57784400

C 1.82062400 3.71525200 -1.63786200

C 2.13504100 -2.91258700 -0.81337400

C 3.32261200 -2.29024000 -0.31773800

C 3.47900800 -0.89586200 -0.38921600

C 4.35758500 -3.13076600 0.37175100

C 0.96305300 -1.71945800 1.99662700

H 3.12928900 -0.27518300 1.79955800

H 3.27138400 1.98962200 1.34784800

H 3.20148500 1.58501600 -0.34703600

H 0.92548800 2.42310200 1.47475500

H -0.66778500 1.90610300 -0.37618200

H 0.62484300 1.16205600 -1.29711700

H 0.19245100 -2.76143900 -1.66512100

H 1.19235600 -1.22649500 -1.74996500

H 1.49554500 5.73017900 0.15587200

H 1.10824900 4.68732000 1.63194200

H 2.71009900 3.12992400 -1.89295400

H 0.99977500 3.32344000 -2.24758700

H 1.99958200 4.74747000 -1.94277600

H 2.00997600 -3.97105100 -0.60375500

H 4.46568100 -0.50639400 -0.15746400

H 3.00197600 -0.39445800 -1.22202200

H 4.88070100 -2.56766100 1.14817200

H 3.93161700 -4.03559800 0.80893100

H 5.11477400 -3.44691400 -0.35714300

H 1.07860300 -1.44321900 3.05130800

H -0.02067000 -2.16606600 1.86196100

H 1.72511900 -2.47950500 1.78865700

Al -2.77962100 -0.30270600 -0.18635800

Cl -3.59700300 -2.26885900 0.29772600

Cl -2.31806400 -0.23621100 -2.33494300

C -3.86103100 1.21455300 0.44510600

H -3.38490400 2.14723500 0.11248500

H -4.82016800 1.17914600 -0.08851200

C -4.12053000 1.27772700 1.96408700

H -4.63283000 0.37871100 2.32051200

H -3.18719200 1.35952500 2.53135800

H -4.74264200 2.13646600 2.24239500

**TSn-3-Al**

E(RB3LYP) -1902,5171163A.U.

1 imaginary frequency -382.12cm^-1^

C -2.39883100 2.21027800 1.04495200

C -3.42159000 1.81261900 0.14359200

C -3.31723400 1.83704100 -1.25440000

C -2.11736900 0.44878100 -1.84493300

C -0.77375800 0.85197600 -1.58727000

C -1.21561300 2.70174600 0.56838100

C -2.60188000 1.96334600 2.52378400

C -2.59412900 -0.89580500 -1.32269000

C -2.04393900 -1.21767600 0.07582400

C -0.51191400 -1.05610200 0.09552900

C 0.01746400 0.14017700 -0.65650900

O 1.26517800 0.41816000 -0.54939500

C -2.48172300 -2.58374600 0.58671100

C -3.17093400 -2.68779600 1.72652800

C -2.11836400 -3.80371900 -0.22462400

C -0.11866000 1.87561300 -2.47822200

H -4.28247300 1.30623500 0.57321500

H -4.21720200 1.57308500 -1.80051400

H -2.76960800 2.65547800 -1.71068800

H -2.41652300 0.62568200 -2.87710400

H -1.09987900 3.05195700 -0.44453400

H -0.37728400 2.87793200 1.23228900

H -2.85071300 0.91850900 2.72970200

H -1.70966300 2.22106800 3.09519900

H -3.42927600 2.57260900 2.89945200

H -3.68641500 -0.94141200 -1.31719600

H -2.25544700 -1.65128000 -2.04168100

H -2.45916900 -0.48032600 0.76564900

H -0.01451100 -1.92270100 -0.35789300

H -0.14748300 -1.01372700 1.12579800

H -3.48813500 -3.65082400 2.11246900

H -3.43777900 -1.81564400 2.31453300

H -2.57964300 -3.77758100 -1.21729100

H -1.03813200 -3.88399000 -0.38078600

H -2.45211400 -4.71423800 0.27520700

H 0.29112200 1.39224000 -3.37249200

H -0.83839200 2.62345600 -2.82466100

H 0.70811800 2.38023500 -1.97947400

Al 2.83141000 -0.22366500 0.19148200

Cl 3.00199600 -2.25332500 -0.61620300

Cl 4.31947200 1.06995100 -0.73788700

C 2.82389000 -0.15994100 2.16039800

H 2.05791900 -0.85111100 2.53775700

H 3.77865100 -0.58908500 2.49308600

C 2.63737000 1.22769800 2.80569600

H 3.40891400 1.93011900 2.47587300

H 1.67008900 1.66986800 2.54273600

H 2.68362200 1.18621700 3.90027600

**TSx-3-Al**

E(RB3LYP) = -1902.517734A.U.

1 imaginary frequency - -379.79cm^-1^

C 1.41415100 -1.88435400 1.51340100

C 2.19589900 -2.84680000 0.93774300

C 3.27677900 -2.47865700 0.09067400

C 3.61451800 -1.15386200 -0.22912000

C 2.34325300 -0.32370700 -1.41858600

C 1.02668300 -0.82674900 -1.19502300

C 2.59353900 1.16493400 -1.22418400

C 1.84301600 1.72571300 -0.00001500

C 0.35426900 1.38067500 -0.10996700

C 0.05133500 -0.02438700 -0.56537300

O -1.16383800 -0.43580900 -0.49634400

C 0.66241800 -2.14424800 -1.80261700

C 2.13173400 3.20465500 0.22771300

C 3.55801300 3.53577300 0.60152000

C 1.20561600 4.16281400 0.14107700

H 4.52785400 -1.01841200 -0.79894800

H 3.66556600 1.35667400 -1.15107600

H 2.24587200 1.68395500 -2.12627200

H 2.23722000 1.21389500 0.88869800

H -0.13904200 2.02772500 -0.84652400

H -0.16218500 1.56251800 0.83613300

H 0.57714800 -2.05425000 -2.89182700

H 1.45871700 -2.87726700 -1.61698100

H -0.27704200 -2.53181400 -1.41318000

H 4.25769300 3.30686600 -0.20879400

H 3.66461800 4.59444900 0.84243700

H 3.87935000 2.95348500 1.47263000

H 1.46492700 5.19965900 0.32657300

H 0.16911100 3.96734800 -0.10282900

H 2.80052800 -0.72109300 -2.32213400

Al -2.79034200 0.00804100 0.24855700

C -4.09637100 -1.38422700 -0.21104000

C -4.38856300 -1.57470900 -1.71318600

H -3.48663800 -1.85376800 -2.26815600

H -5.13318800 -2.35891900 -1.89355700

H -4.77158100 -0.65527600 -2.16678500

H -3.75047000 -2.33249900 0.22253100

Cl -2.34225900 0.14946500 2.39286400

Cl -3.26633900 2.00026000 -0.52553800

H -5.03063900 -1.15168800 0.31684500

H 0.50794100 -2.14332400 2.04818400

H 1.71145400 -0.84840900 1.56306400

H 3.49384600 -0.42115100 0.56058400

C 1.86121600 -4.31467800 1.08677200

H 1.74022300 -4.80026500 0.11456900

H 2.66644600 -4.83581000 1.61329300

H 0.94249700 -4.45543100 1.65703700

H 3.78604400 -3.27970600 -0.43927000
